# CD4 is expressed on a heterogeneous subset of hematopoietic progenitors, which persistently harbor CXCR4 and CCR5-tropic HIV proviral genomes in vivo

**DOI:** 10.1371/journal.ppat.1006509

**Published:** 2017-07-21

**Authors:** Nadia T. Sebastian, Thomas D. Zaikos, Valeri Terry, Frances Taschuk, Lucy A. McNamara, Adewunmi Onafuwa-Nuga, Ryan Yucha, Robert A. J. Signer, James Riddell IV, Dale Bixby, Norman Markowitz, Sean J. Morrison, Kathleen L. Collins

**Affiliations:** 1 Program in Cellular and Molecular Biology, University of Michigan, Ann Arbor, Michigan, United States of America; 2 Medical Scientist Training Program, University of Michigan, Ann Arbor, Michigan, United States of America; 3 Department of Microbiology and Immunology University of Michigan, Ann Arbor, Michigan, United States of America; 4 Division of Infectious Disease, Department of Internal Medicine University of Michigan, Ann Arbor, Michigan, United States of America; 5 Howard Hughes Medical Institute, University of Texas Southwestern Medical Center, Dallas, Texas, United States of America; 6 Children’s Research Institute, University of Texas Southwestern Medical Center, Dallas, Texas, United States of America; 7 Department of Pediatrics, University of Texas Southwestern Medical Center, Dallas, Texas, United States of America; 8 Division of Oncology, Department of Internal Medicine, University of Michigan, Ann Arbor, Michigan, United States of America; 9 Division of Infectious Diseases, Henry Ford Hospital, Detroit, Michigan, United States of America; Universitätklinikum Heidelberg, GERMANY

## Abstract

Latent HIV infection of long-lived cells is a barrier to viral clearance. Hematopoietic stem and progenitor cells are a heterogeneous population of cells, some of which are long-lived. CXCR4-tropic HIVs infect a broad range of HSPC subtypes, including hematopoietic stem cells, which are multi-potent and long-lived. However, CCR5-tropic HIV infection is limited to more differentiated progenitor cells with life spans that are less well understood. Consistent with emerging data that restricted progenitor cells can be long-lived, we detected persistent HIV in restricted HSPC populations from optimally treated people. Further, genotypic and phenotypic analysis of amplified *env* alleles from donor samples indicated that both CXCR4- and CCR5-tropic viruses persisted in HSPCs. RNA profiling confirmed expression of HIV receptor RNA in a pattern that was consistent with in vitro and in vivo results. In addition, we characterized a CD4^high^ HSPC sub-population that was preferentially targeted by a variety of CXCR4- and CCR5-tropic HIVs in vitro. Finally, we present strong evidence that HIV proviral genomes of both tropisms can be transmitted to CD4-negative daughter cells of multiple lineages in vivo. In some cases, the transmitted proviral genomes contained signature deletions that inactivated the virus, eliminating the possibility that coincidental infection explains the results. These data support a model in which both stem and non-stem cell progenitors serve as persistent reservoirs for CXCR4- and CCR5-tropic HIV proviral genomes that can be passed to daughter cells.

## Author summary

People who are effectively treated with antiretroviral medication harbor persistent forms of HIV that are integrated into the cellular genome. While HIV is cytopathic to most cells, transcriptionally silent, latent forms do not express toxic HIV gene products and can survive in the host for years. When conditions change, the latent virus can be activated to reinitiate infection. Because of the capacity for virus to spread, cure of HIV will require that we identify and eradicate all cells harboring functional HIV provirus. CD4^+^ T cells are abundant and easily identified as harboring proviral genomes. However, rare cell types that express HIV receptors, such as bone marrow hematopoietic progenitor and stem cells can also be infected by the virus potentially serving as barriers to cure strategies. We found that HIV can infect and persist in progenitor sub-types that were previously thought to be short lived, which expands the types of cells that can support reservoir formation. In addition, we found that HIV can spread by proliferation and cellular differentiation without the need for viral gene expression and virion production that could reveal the infection to the immune system. A deeper understanding of viral reservoirs is critically important for developing strategies that will succeed in viral eradication.

## Introduction

Long term combination anti-retroviral therapy (cART) blocks viral spread in vivo but is not curative, as plasma virus rebounds after cART interruption. Sequence analysis of residual circulating and rebounding virus in HIV^+^ patients indicates that virions likely come from the activation of latent provirus that had been archived since before the initiation of therapy rather than from low-level replication and spread of cART-resistant virus [1, 2].

HIV enters cells via HIV Env interacting with CD4 plus a co-receptor, usually CCR5 or CXCR4. CXCR4-utilizing viruses differ from those that utilize CCR5 in their ability to infect stem cells that can engraft and generate multiple lineages in a mouse xenograft model [3]. In contrast, CCR5-tropic viruses infect HSPCs that are restricted in their capacity to differentiate [3]. Recently, Nixon and colleagues elegantly demonstrated that myeloid progenitors, including common myeloid progenitors (CMPs) and granulocyte/monocyte progenitors (GMPs), express CCR5 and can be infected by CCR5-tropic HIV in vitro and in a humanized mouse model [4].

Based largely on patterns of hematopoiesis that occur following transplantation, hematopoietic progenitors, such as those targeted by CCR5-tropic HIVs, were thought to be short-lived in vivo [3–5]. However, in situ tagging experiments in mice have recently found that non-stem cell progenitors make an enduring contribution to native hematopoiesis in adults through successive recruitment of thousands of clones, each with a minimal contribution to mature progeny [6–8]. Consistent with this, non-stem cell myeloid progenitors such as GMPs were found to persist in people with aplastic anemia despite dramatic losses of stem cells [6]. Thus, a large number of long-lived progenitors, rather than classically defined Hematopoietic stem cells (HSCs), may be the main drivers of steady-state hematopoiesis during adulthood [7, 8].

Here, we provide evidence that non-stem cell hematopoietic progenitors harbor CCR5-tropic HIVs for years in optimally treated people, providing new evidence that non-stem cell progenitors are long-lived in people without evidence of bone marrow disease and can potentially serve as reservoirs of HIV. We also demonstrate that CD4^high^ HSPC subsets that we show include multi-potent progenitors (MPPs) are preferentially targeted by both HIV subtypes in vitro. Moreover, we provide in vivo evidence that infected HSPCs can differentiate into multiple lineages that harbor provirus. These data expand our understanding of HIV infection and hematopoiesis by demonstrating that in addition to stem cells, intermediate progenitor cells potentially provide an enduring reservoir for CCR5- and CXCR4-tropic HIV proviral genomes.

## Results

### Isolation of stem-cell-enriched and depleted HSPC populations from patient samples

**Fig 1:**
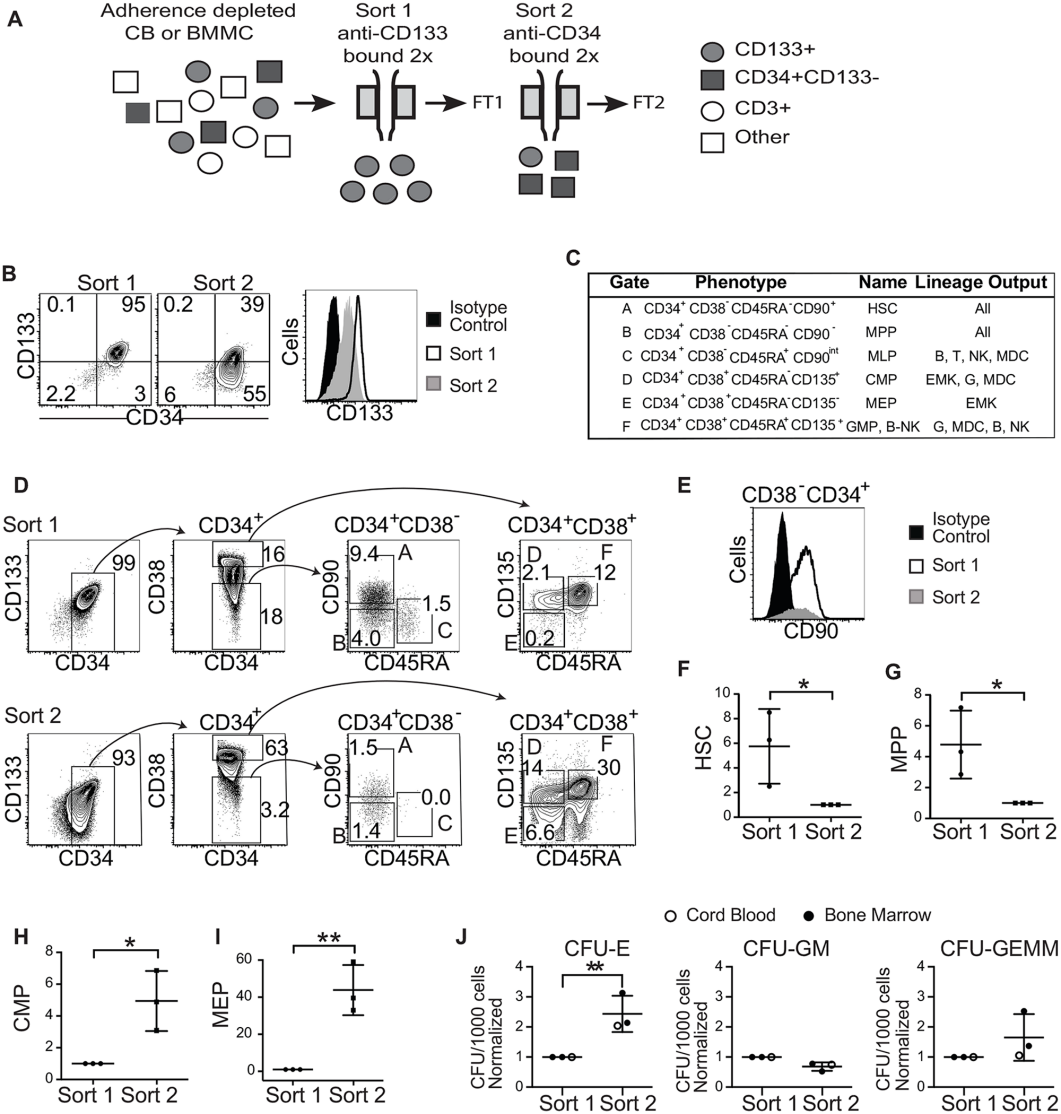
Sort 2 HSPCs are depleted for HSCs. A. Diagrammatic representation of HSPC purification. FT, column flow-through; CB, cord blood; BMMC, bone marrow mononuclear cells. B. Representative flow cytometric analysis of HSPC samples from an HIV^+^ individual. C. Gates for each HSPC population phenotype and lineage output according to Doulatov *et al* [9]. (HSC, hematopoietic stem cell; MPP, multipotent progenitor; MLP, multilymphoid progenitor; CMP, common myeloid progenitor; MEP, megakaryocyte/erythrocyte progenitor; GMP, granulocyte/monocyte progenitor; B-NK, B and NK cell progenitor; MDC, macrophage and dendritic cell; EMK, erythroid and megakaryocyte; G, granulocyte.) D. Representative flow cytometric analysis of differentiation markers expressed on bone marrow HSPCs purified as described in A. For the two right-most panels, numbers indicate percentage of total CD34^+^ events from each sort falling into that gate. E. Flow cytometric plot comparing relative numbers of HSCs (CD34^+^CD38^-^ cells that are also CD90^+^) in Sort 1 versus Sort 2. F-I. Summary graph showing the relative frequency of the indicated progenitor in each sort, *n* = 3 uninfected donors. To facilitate comparison, results were normalized to Sort 2 (F and G) or Sort 1 (H—I). Mean ± standard deviation is indicated. J. Summary plots of methylcellulose colony formation assays from three uninfected donors. Mean ± standard deviation is indicated. Colony forming unit (CFU)-E, erythroid; CFU-GM, granulocyte/macrophage and CFU-GEMM, multilineage. (*p<0.05 and **p<0.01, 2-tailed Student’s t-test).

To better understand the types of hematopoietic stem and progenitor cells (HSPCs) that are infected by HIV in vivo, we developed an approach to efficiently isolate HSPC populations enriched (Sort 1) or depleted (Sort 2) for stem cells (Fig 1A and 1B). Compared to Sort 1 cells, Sort 2 cells expressed lower levels of CD133 (Fig 1B) and were depleted for hematopoietic stem cells (HSCs) and multi-potent progenitors (HSC/MPPs) (Fig 1C–1G). Conversely, Sort 2 cells were enriched for more restricted progenitors (common myeloid progenitors (CMPs) and megakaryocyte/erythrocyte progenitors (MEPs) (Fig 1H and 1I)[9]. Enrichment of MEPs in Sort 2 samples was confirmed using methylcellulose colony formation assays (Fig 1J).

To develop a better understanding of which HSPCs harbor HIV in vivo, we obtained samples from 47 HIV-infected donors, including two that had been initially treated during acute infection. All donors were on therapy with undetectable viral loads for least six months. A 20 ml bone marrow sample and 100 ml of peripheral blood were collected from each donor. HSPCs were isolated from adherence depleted bone marrow mononuclear cells in two steps as described in Fig 1. From 20 cc of bone marrow, we obtained ~2.5x10^6^ total HSPCs per donor. For 41 of 47 donors, we obtained adequate aspirates and the purified HSPCs met our criteria of having <1% CD3^+^ T cell contamination and >80% CD34^+^ or CD133^+^ cells. The mean purity of included samples was approximately 94% CD133 for Sort 1 and 90% CD34 for Sort 2 (Table 1, S1 and S2 Tables).

**Table 1:** Donor characteristics.

HSPC proviral DNA	Positive	Below the limit of detection
Number of donors	24(59%)	17 (41%)
Number treated since acute infection	1 (50%)	1 (50%)
Male	21	15
Female	3	2
Black	3	4
White	21	13
Non-Hispanic	22	17
Hispanic	2	0
CD4 count (mean)	849	678
CD4 count (range)	215–2060	308–1112
Viral load	<48	<48
Duration of suppression, years (mean)	3.6	4.4
Range of suppression, years	0.5–9.8	0.6–11.1
Mean Sort 1 purity (% CD133, % CD3)	94.7, 0.31	93.1, 0.32
Mean Sort 2 purity (% CD34, % CD3)	90.5, 0.21	89.9, 0.22
Mean number of cells screened (per donor)	975,949	661,965
Range of cells screened	167,375–3,208,600	83,070–1,300,000

DNA was isolated from each sample and multiplex single genome amplification (SGA) polymerase chain reaction (PCR) was used to amplify *gag* and *env* amplicons or near full-length genomes. For each donor, we selected a primer pair combination that most efficiently amplified HIV sequences from peripheral blood mononuclear cell (PBMC) DNA prior to testing HSPC samples. After analyzing at least 80,000 cells from all samples that met our purity criteria, we determined that most donors (n = 24, 59%) had detectable HIV provirus in HSPCs. More cells were screened in the positive group than in the undetectable group (975,959 versus 661,965) but that difference and the level of sample purity between the two groups were not statistically significant (Table 1). Further, the timing of HAART was not a significant factor in our ability to detect provirus; one of two donors treated since acute infection had detectable HSPC-associated provirus and provirus was present in long term suppressed patients (up to 9.8 years, Table 1). The overall mean frequency of provirus in HSPCs was 2.4 copies per million cells based on the number of positive 1^st^ round PCR reactions (82) set up at limiting dilution that produced a *gag* and/or *env* amplicon out of the total number of cells assayed (35 million). For individual donors, the frequency ranged from <1 per 1.3 x10^6^ cells to 18 copies per 10^6^ cells.

#### Frequency of replication competent virus

**Fig 2:**
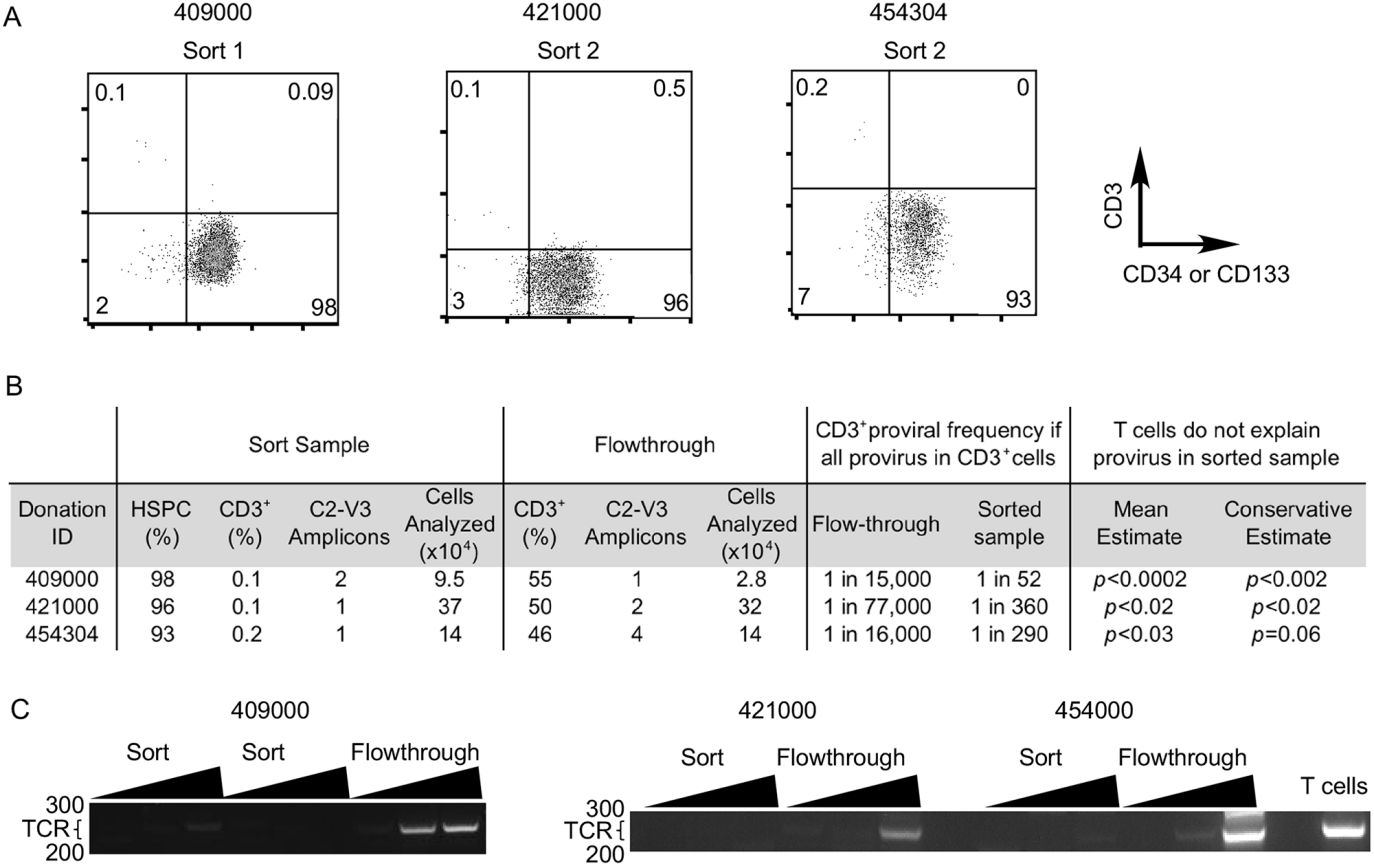
Near-full-length HIV provirus in HSPCs preparations is unlikely to be from T cell contamination. A. Flow cytometric analysis of HSPCs from three donors that harbored near full-length genomes. Numbers indicate the frequency of events falling into each quadrant. CD133 or CD34 were assessed for Sort 1 or Sort 2 respectively. B. Summary table showing data for Fisher’s exact test, which compares calculated frequencies of provirus assuming all provirus originates from T cells. PCRs were performed at limiting dilution. Significantly discrepant frequencies in sorted and flow-through samples indicate T cells are an unlikely source of provirus in sorted samples. Conservative estimate compares the top of the 95% confidence interval for the calculated infection rate for flow-through CD3^+^ T cells with the bottom of the 95% confidence interval for the calculated infection rate for CD3^+^ T cells in the sorted HSPCs. C. Agarose gel analysis of rearranged T cell receptor gene PCR. 50, 100 or 200 cell equivalents of DNA from the first round PCR reaction that yielded each provirus were added to each reaction. For “T cells”, 200 cell equivalents of DNA from purified CD4^+^ T cells were added to the reaction. The expected size of the amplicons is 250–300 bp. Numbers to the left of each gel indicate the location of molecular weight markers.

To assess the frequency of replication competent virus amongst the proviral DNA that could be amplified from HSPCs, we utilized primers that generated near full–length (approximately 9000 base pair) amplicons. From a total of 14 reactions that generated amplicons using these primers (S2 Table), subsequent analysis identified four with intact open reading frames and cis elements (S3 Table), suggesting that approximately 30% of proviral genomes in HSPCs are likely to be functional. All amplicons were directly sequenced from the purified gel band and screened to ensure they were not identical to sequences from other donors or molecular clones used in the lab (S1 Fig). The amplified near full-length proviral genomes came from highly purified HSPCs (Fig 2A) and were unlikely to have been derived from CD3^+^ T cells (see below and Fig 2).

### CD3^+^ T cells are an unlikely source of HIV DNA in HSPC samples

To rule out T cells as a source of HIV DNA in HSPC samples, we eliminated all HSPC samples with >1% contaminating CD3^+^ cells and all samples included in our final analysis contained <0.52% CD3^+^ cells (S1 and S2 Tables).

In addition, we used a previously published statistical method that takes into account HIV genome frequency in sorted and flow-through samples, assigning a *p* value to indicate the likelihood that HIV DNA in HSPC samples came from T cells [10]. This analysis is shown in detail in Fig 2. Briefly, we carefully assessed the frequency of CD3^+^ T cells and provirus in both the sorted sample and in the flow-through sample. Then, we compared the frequencies assuming that only CD3^+^ T cells account for all provirus. As shown in Fig 2B, the frequency of infected CD3^+^ T cells would have to have been much higher in the sorted sample than in the flow-through to account for the provirus in the sorted HSPC samples (e.g. 1 in 52 versus 1 in 15,000 for donor 409000). This difference is assigned a *p* value that takes into account 95% confidence intervals and only samples with *p*<0.05 were included in our final analysis.

Consistent with our conclusion that HIV DNA from Sort 1 and 2 came from HSPCs and not CD3^+^ T cells, we observed no correlation between proviral frequency in the samples and the frequency of contaminating CD3^+^ cells (S2 Fig). In addition, rearranged T cell receptor PCR assays were performed to confirm that near-full-length genomes from HSPC DNA samples were unlikely to have originated from T cells (Fig 2C). Similar results were obtained from donor 413402, which was screened by PCR because too few cells were available to accurately assess this sample by flow cytometry (S4 Table, S3 Fig). [The caveats for the rearranged TCR PCR assay are that it is not quantitative and it is associated with non-specific background bands that limit the amount of DNA that can be added to the reaction. These non-specific bands arise in all samples, including negative control HEK 293 cells, and are not related to TCR based on sequencing analysis. Because of these limitations, the statistical analysis we described in Fig 2 provides a more robust and quantitative assessment.]

### Both CCR5 and CXCR4-tropic HIVs persist in bone marrow HSPCs

Based on initial results that CCR5-tropic HIVs infect non-stem-cell progenitors that were originally believed to be short lived, we expected to mainly observe CXCR4-tropic virus in HSPC preparations. To assess this, we examined *env* amplicons available to study from a subset of 19 donors from the overall cohort. As summarized in Tables 2 and 3, we isolated a total of 52 *env* C2-V3 amplicons. Each amplicon was assigned a genotype using the indicated co-receptor prediction software (Table 3). 16 amplicons from 8 donors were predicted to be CXCR4-tropic, including three near-full-length genomes with full open reading frames and cis elements (S3 Table). Unexpectedly, we also isolated a total of 36 amplicons from 17 donors that were predicted to be CCR5-tropic, including one near-full-length genome with full open reading frames and cis elements (S3 Table). Overall, the genotopyes of *env* amplicons from HSPCs closely matched those from peripheral blood mononuclear cells for each donor (Table 3).

**Table 2:** Summary of donor *env* amplicons in Sort 1 and 2 HSPCs.

Number of donors with C2-V3 *env* amplicons	19
Number C2-V3*-env* amplicons amplified from HSPCs	52
Donors with CXCR4-tropic Env in HSPCs	8
Donors with CCR5-tropic Env in HSPCs	17
Donors with proviral genomes of both tropisms in HSPCs	6
Donors with provirus in stem cell enriched fraction (Sort 1)	14
Donors with provirus in progenitors depleted for stem cells (Sort 2)	11
Donors with provirus in both HSPC populations	6

**Table 3:** Analysis of *env* amplicons isolated from HIV^+^ donors.

Donor ID (% purity) (S1, S2)	HSPC Sort 1 (S1)				HSPC Sort 2 (S2)				PBMC		
	*env* amplicons				*env* amplicons				*env* amplicons		
	#	FPR	Geno^b^	Pheno^c^	#	FPR	Geno	Pheno	#	FPR	Geno
409000(98, 98)	1****w 1****w 1*w	61 0.7 0.7	R5 X4/Dual X4/Dual		ND				17 11	38–61 0.7	R5 X4/Dual
413402 (94, 91)	1*	1.7	X4/Dual	Dual	ND				6	1.7	X4/Dual
414000 (95, 85)	1*	57	R5	R5	ND				5	57	R5
415000 (95, NA)	1**	71	R5	R5	NA				20	43–83	R5
419000 (95, NA)	1***	89	R5		NA				21	22–99	R5
420000 (99, 92)	1**** 1****w 1**** 1****w	19 73 0.7 8.1	R5 R5 X4/Dual X4/Dual	R5 Dual	ND				11 7	14–73 0.5–1.7	R5 X4/Dual
421000 (99, 96)	1***w 2***	7.8 7.4,7.8	X4/Dual X4/Dual	X4	1**w	7.8	X4/Dual		4 5	24–94 4.7–9.6	R5 X4/Dual
426000 (97, 92)	ND				4**** 2****w 1****w	29–60 20–31 4.8	R5 R5 X4/Dual		10 1	20–38 3.2	R5 X4/Dual
428408 (95, 86) (91, 91)	2**** 1****	83,84 1.3	R5 X4/Dual		1**w	1.3	X4/Dual		22 16	30–100 0.7–6.8	R5 X4/Dual
431000 (93, 89)	2**** 1****	75 79	R5 R5	R5 R5	ND				8	38–90	R5
432000 (98, 99)	1**** 1****	17 2.8	R5 X4/Dual	X4	1**** 1****	2.8 3.4	X4/Dual X4/Dual	NF	1 8 1 11 4	49 3.4–8.5 74 2.8–4.7 3.4–8.5	R5 X4/Dual R5 X4/Dual X4/Dual
434423 (92, 89) (94, 84)	1**	38	R5		1**	46	R5		18	38–82	R5
435412406 (99, 99) (98, 83) (95, 94)	1** 2**** 1** 1**w	83 87 89 45	R5 R5 R5 R5	R5 R5 R5	1**	83	R5	R5	103	42–99	R5
436000 (93, 85)	ND				2**	31	R5	R5	8	31–85	R5
437000 (94, 92)	1**	55	R5				ND		8	12–55	R5
449000 (90, 95)	1*	100	R5		1**	81	R5		5	59–99	R5
453000 (96, 83)	ND				1***	74	R5	R5	6	41–86	R5
454304 (92,93)	ND				1* 1*w	31 9.6	R5 X4/Dual		24 1	11–97 6.8	R5 X4/Dual
456000 (93,90)	ND				3****	13–39	R5		10 2	10.5–39 7.4, 9.2	R5 X4/Dual

^#^ indicates the number of amplicons of that type isolated from each donor.

^b^genotypic prediction of co-receptor usage by Geno2pheno using a false positive rate (FPR) cutoff of ≤10% [36, 37].

^c^phenotypic analysis of co-receptor usage (see Table 4).

“w” indicates amplicons from whole genome PCR and black text indicates amplicons from multiplex PCR reaction.

Asterisks reflect the likelihood that CD3^+^ T cells contamination was responsible for the amplicon

*p < 0.05 by (1) only

**p < .05 by (1) and (2)

***p < .01 by (1) and (2)

****p < .001 by (1) and (2) where (2) is using the more conservative assessment (Fig 2) [10].

Abbreviations: HSPC, hematopoietic stem and progenitor cells; PBMC, peripheral blood mononuclear cells; NA, not analyzed due to purity concerns; R5, CCR5; X4, CXCR4; ND, not detectable; NF, non-functional.

Each set of three non-zero numbers in the donor name represents an independent donation.

Because *env* genotype prediction tools are not always reliable, we confirmed Env tropism with a phenotypic assay. For this analysis, we used either HSPC-derived full-length Env or a non-HSPC-derived Env with identical nucleotide or amino acid V3 region from the same donor as available (Table 4). A phenotypic assay utilizing 3T3 cells expressing CD4 and individual chemokine receptors [11] was used for this assessment. This assay confirmed the tropism of ten CCR5-tropic Envs, four CXCR4/dual tropic Envs and demonstrated that one Env was not functional. The isolation of HIV encoding Envs of both tropisms from HSPCs suggests either that CCR5-tropic Envs unexpectedly target HSCs or that restricted progenitor cells targeted by CCR5-tropic viruses survive longer in vivo than expected.

**Table 4:** Summary of Env phenotypes with V3 regions matching HSPC-associated *env* sequences.

Donor ID	Source of full length Env Amplicon	Source of Matching V3 Amplicon	FPR	Genotype	Phenotype
413402	PBMC	Sort 1	1.7	X4/Dual	Dual
414000	PBMC	Sort 1	57	R5	R5
415000	HSPC	Sort 1	71	R5	R5
420000	PBMC	Sort 1	19	R5	R5
420000	PBMC	Sort 1	0.7	X4/Dual	Dual
421000	HSPC	Sort 1	7.8	X4/Dual	X4
431000	Sort 1 FT	Sort 1	75	R5	R5
431000	PBMC	Sort 1	79	R5	R5
432000	HSPC	Sort 1	2.8	X4/Dual	X4
432000	HSPC	Sort 2	2.8	X4/Dual	NF
435412406	PBMC	Sort 1	83	R5	R5
435412406	PBMC	Sort 1	87	R5	R5
435412406	PBMC	Sort 1	89	R5	R5
436000	PBMC	Sort 2	31	R5	R5
453000	PBMC	Sort 2	74	R5	R5

Abbreviations: FT, flow-through; NF, non-functional Env; FPR, false positive rate from Geno2pheno.

Each set of three non-zero numbers in the donor name represents an independent donation.

### Evidence that restricted progenitors targeted by CCR5-tropic viruses persist in vivo

To better understand whether CCR5-tropic viruses might target restricted progenitors that persist longer than expected, we asked whether provirus could be detected in Sort 2, which contained restricted progenitors that were unlikely to be stem cells. Interestingly, we found no significant difference in the number of donors with amplicons in Sort 1 versus Sort 2 subsets [14 donors had amplicons isolated from Sort 1 and 11 had amplicons isolated from Sort 2, (Tables 2 and 3)]. The mean frequency of *env* amplicons was higher in Sort 1 than Sort 2 but this difference did not achieve significance. (The mean frequency was four copies per million cells for Sort 1 versus two copies per million cells for Sort 2, *p* = 0.06.) None of the amplicons isolated from Sort 2 were identical to those from Sort 1, indicating that independent infection of restricted progenitors rather than differentiation of infected stem cells explains the presence of provirus in this population. In sum, these results suggest that non-stem cell restricted HSPCs can be infected by HIV and endure for at least the period of effective antiretroviral treatment.

### CD4, CCR5 and CXCR4 RNA are expressed in a variety of HSPC subsets

**Fig 3:**
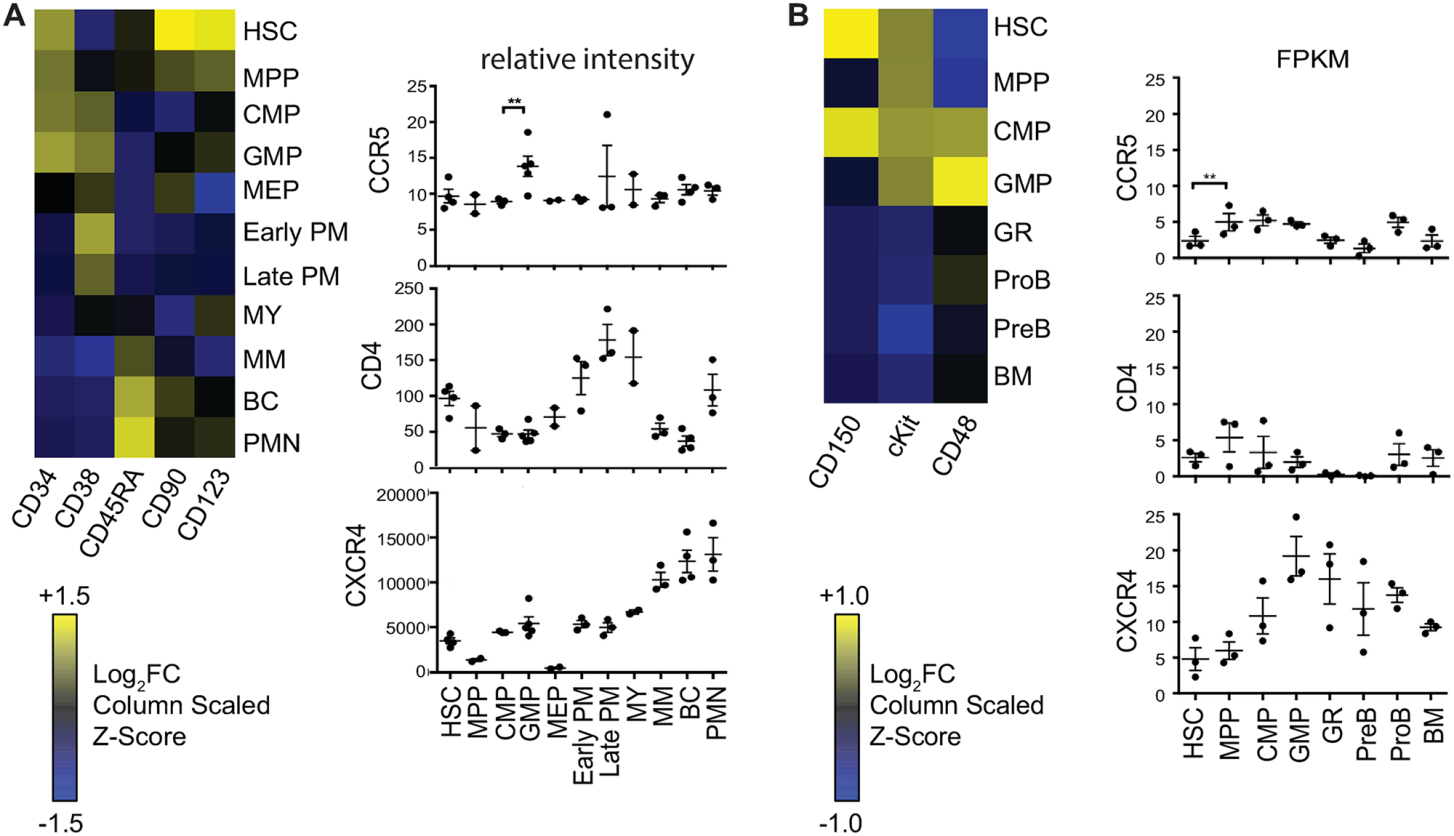
Expression of HIV receptors in progenitor cells. A. Affymetrix array data of gene expression in human bone marrow stem and differentiated cell types extracted from a published data set [12] accessed via the NCBI Gene Expression Omnibus database (GSE42519). Left panel, heat map representing the z-score (scaled for each column) of the log transformed gene expression data. Right panel, relative expression levels of HIV receptors in each subset. The original microarray data went through background correction, normalization, and log transformation via the RMA method. For the right panel, data was converted from log transformed to linear. B. RNA seq analysis of mouse bone marrow and differentiated cell types. Left panel, heat map representing the z-score (scaled for each column) of the log transformed gene expression data. Right panel, relative expression levels of HIV receptors in each subset. Mean ± SEM is shown. (Abbreviations: FC, fold change; HSC, hematopoietic stem cell; MPP, multipotent progenitor; CMP, common myeloid progenitor; GMP, granulocyte-monocyte progenitor; MEP, megakaryocyte-erythrocyte progenitor; PM, promyelocyte; MY, myelocyte; MM, metamyelocytes; BC, band cell; PMN, polymorphonuclear cells; GR, granulocyte; ProB, pro-B cell; PreB, pre-B cell; BM, unfractionated bone marrow. FPKM, Fragments Per Kilobase Of Exon Per Million Fragments Mapped) (*p<0.05, **p<0.01, ***p<0.001, ****p<0.0001, 2-tailed unpaired t test).

The result that HSPCs depleted of HSCs harbor HIV provirus that persists in optimally treated people as well as the finding that CCR5-utilizing virus persists in HSPCs was unexpected; therefore, we pursued additional evidence to better understand this finding. First, we assessed expression of HIV receptors in HSPC subsets. To accomplish this, we used a publicly available microarray dataset of RNA expression in human bone marrow HSPCs [12] and used established markers to purify murine bone marrow HSPCs for an RNA-seq analysis to profile expression of HIV receptors in HSPC subtypes. After confirming that progenitor subsets from each species expressed the expected developmentally appropriate set of genes (Fig 3) we found that both approaches yielded similar results. As shown in Fig 3, both revealed very low CCR5 expression in HSCs with higher expression in some restricted hematopoietic progenitor sub-types. These results agree with published studies showing low or no expression of CCR5 protein by HSC-enriched cells [3, 13] with more expression of CCR5 protein in restricted hematopoietic progenitor sub-populations [4, 13]. In addition, both approaches showed that CXCR4 and CD4 RNA were expressed by HSCs and several other progenitor populations (Fig 3). Based on this analysis, CXCR4-tropic viruses are predicted to target a wide range of progenitor subsets including HSCs whereas CCR5-tropic Envs are more likely to target restricted HSPC subsets such as GMPs.

### Full length and transmitter/founder CCR5-tropic HIVs target restricted hematopoietic progenitors that are unlikely to be stem cells

**Fig 4:**
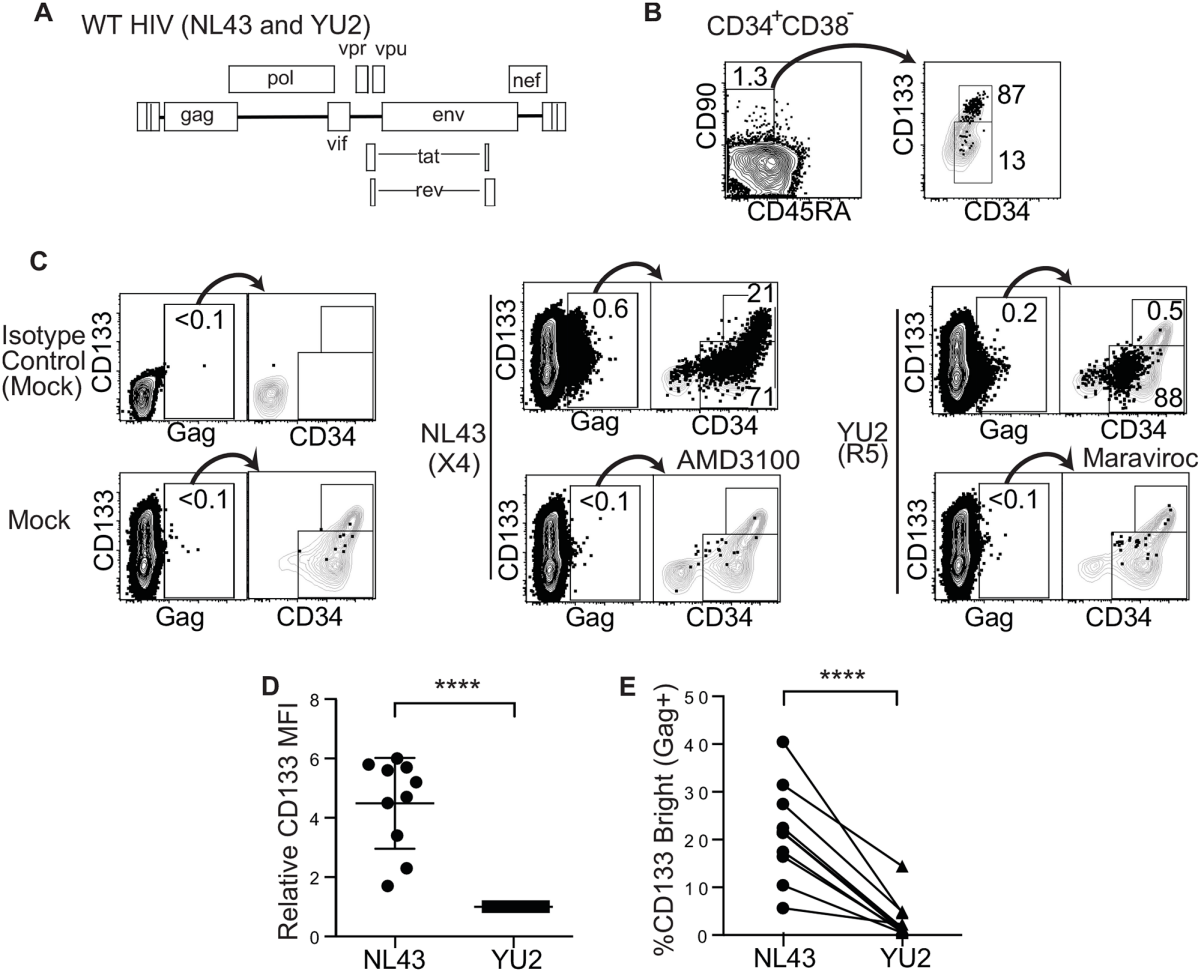
A. Schematic of full-length HIV used in C-E. B. Flow cytometric plots of CD133-sorted cord blood HSPCs cultured for 7 days and stained as in Fig 1D. Cells gated in left plot were overlaid on the total live population on the right. C. Representative flow cytometric plots of cord blood-derived CD133-sorted cells expanded for four days, infected with the indicated virus and harvested 2 days post-infection. Gag ^+^ cells are overlaid onto CD34 versus CD133 plots for the total live cell population and the percentage of Gag^+^ cells in the CD133^high^ and CD133^low^ regions is indicated in the overlay. D. Summary graph of relative CD133 expression by mean fluorescence intensity (MFI) on HSPCs infected with the indicated HIV. Results were normalized to those for YU-2 infected cells for each experiment. E. Summary graph showing the frequency of Gag^+^ cells falling into CD133^bright^ gate. Data was compiled from four cord blood experiments; mean ± standard deviation is indicated (****p < 0.0001, 2-tailed paired t-test).

**Fig 5:**
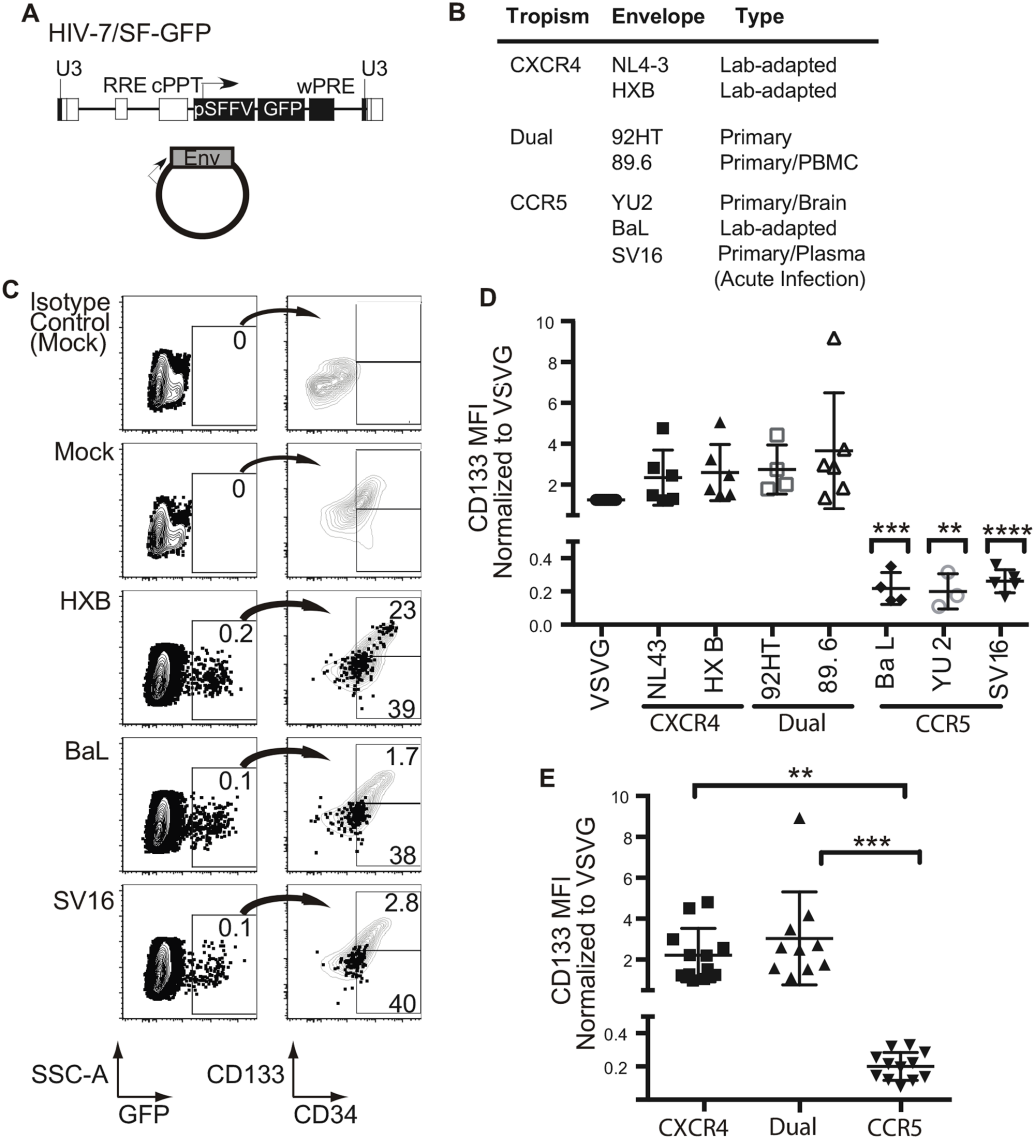
Targeting of intermediate progenitors by CCR5-tropic Envs is a conserved property extending to a transmitted/founder virus. A. Schematic of HIV-7/SF-GFP construct and HIV envelope plasmid used to construct pseudotyped viruses used in C-E. B. Summary table of envelope proteins used to pseudotype HIV-7/SF-GFP virus. C. Representative flow plots of cord blood-derived CD133-sorted cells expanded for four days, transduced with the indicated virus and harvested 3 days post-infection for flow cytometric analysis. In each right panel, GFP^+^ cells were overlaid onto plots of the total cell population and the percentage of GFP^+^ cells in the CD133^high^ and CD133^low^ regions is indicated. Gates were determined based on isotype control antibody staining (top panel). D. Summary graph of CD133 MFI for experiments performed as in C. Results are compiled from 11 cord blood experiments. Mean ± standard deviation is indicated; *n*≥3 for each envelope. 2-tailed Student’s t-test indicates significance for each HIV envelope with respect to VSV-G (**p < 0.01,***p < 0.001, ****p < 0.0001). E. Data from (D) compiled by tropism. Mean ± standard deviation is indicated; one-way ANOVA, *p* = 0.0002, with Tukey’s Multiple Comparisons Test indicated (**p < 0.01 and ***p < 0.001).

In prior studies, we used pseudotyped lentiviral reporter constructs to examine differential targeting of HSPCs by CCR5 and CXCR4 and it remained possible that full length, wild type HIVs target cells differently. To examine this question, we compared HIV infection of HSPCs by two wild type viruses, NL4-3 (CXCR4-tropic) and YU-2 (CCR5-tropic) (Fig 4A). After demonstrating that CD133^bright^ cell populations contain the majority of HSCs based on CD38, CD45RA and CD90 staining (Fig 4B), we used the level of CD133 staining to assess HIV infection of HSCs. As shown in Fig 4C, full length HIVs demonstrated the same pattern as previously observed using pseudotyped lentiviral vectors; CCR5-tropic YU2 infected a restricted pattern of progenitors depleted of stem cells whereas NL4-3 targeted a wide range of progenitors, including those likely to be stem cells. (Maraviroc and AMD3100 appropriately inhibited entry via CCR5 and CXCR4 respectively, Fig 4C, lower panels.) Correspondingly, on average, we measured about 4.5 times more CD133 on HSPCs infected by NL4-3 than those infected with YU2 (Fig 4D and 4E). Further, the same pattern was observed using a lentiviral construct (HIV-7/SF-GFP) pseudotyped with additional Env proteins including one from a CCR5-tropic transmitted/founder virus [SVPB16 (SV16)][3, 14, 15 (Fig 5). In sum, consistent with prior results, CCR5-tropic viruses consistently demonstrated a restricted pattern of infection of more differentiated progenitors that contrasts with the wide range of progenitors targeted by CXCR4-tropic and VSV-G-pseudotyped viruses. Confirmation that a wide range of CCR5-tropic HIVs are restricted to non-stem cell HSPCs suggests that the CCR5-tropic HIV we detected in stem cell–depleted HSPC populations from patients likely came from more restricted progenitors that survived longer than previously appreciated and that these cells might also serve as long lived cellular reservoirs of HIV.

### CCR5- and CXCR4-utilizing viruses target a separable population of multipotent HSPCs that have high levels of CD4

**Fig 6:**
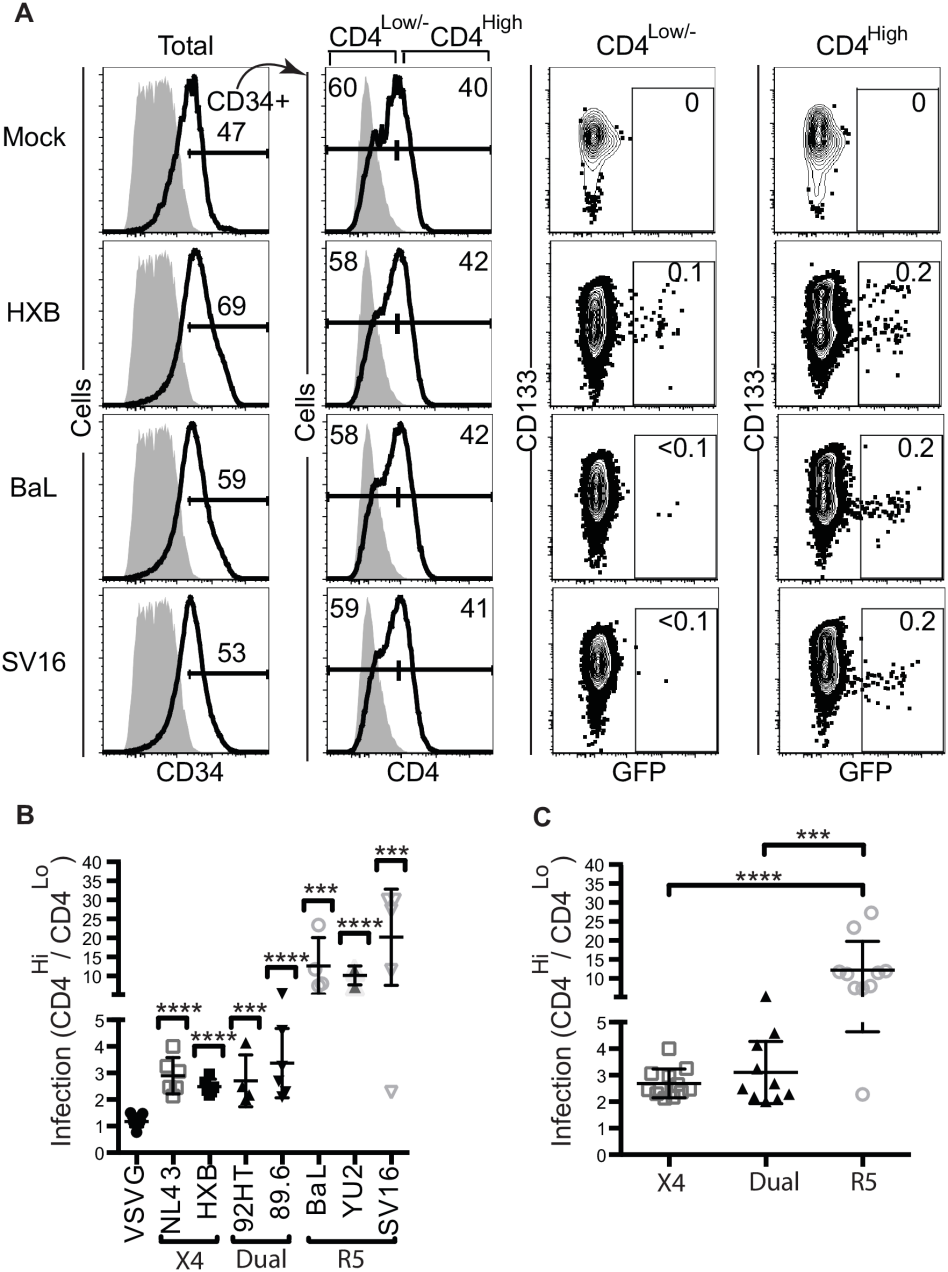
HSPCs with greater CD4 expression are preferentially infected by HIV Envs. A. Representative flow cytometry plots and gating strategy for cord blood-derived CD133-sorted cells infected with virus containing the indicated envelope protein and harvested 3 days post-infection. Gating for CD4 was determined by the inclusion of 1% of cells stained with an isotype control antibody (gray). For GFP plots, numbers indicate the percentage of GFP^+^ events. B. Summary graphs depicting the ratio of infected cells in CD4^high^ versus CD4^low/-^ subsets of cord blood-derived HSPCs infected and analyzed as in part A. For SV16, two replicates had 0.0% infection in the CD4^low/-^ gate leading to an undefined ratio, so 30.0 was used as a conservative estimate of the ratio. Data from 11 uninfected cord blood experiments. Mean ± standard deviation is indicated; n≥3 for each envelope. Results were compared to infection by VSV-G pseudotyped viruses and *p* values were determined using 2-tailed Student’s t-test (***p<0.001, ****p<0.0001). C. Data from B but compiled by tropism. Mean ± standard deviation is indicated; one-way ANOVA, *p*<0.0001, with Tukey’s Multiple Comparisons Test indicated (***p<0.001 and ****p<0.0001).

The HIV receptor, CD4, is usually required for infection and is expressed on CD34^+^ HSPCs, although at low levels compared to CD4^+^ T cells [16, 17]. If the relative level of CD4 expression on HSPCs determined susceptibility of HSPCs to infection, then CD4 expression would serve as an indicator of the subtypes of HSPCs potentially targeted by HIV. To examine this question, we treated HSPCs with a GFP-expressing lentiviral vector pseudotyped with CCR5- or CXCR4-tropic Env proteins (Fig 6A and 6B) and assessed CD4 levels on the GFP^+^ transduced cells. We observed that HSPCs within a CD4^high^ flow cytometric gate displayed 2–30 times greater infection than CD4^low/-^ cells (Fig 6A–6C). The increased infection of CD4^high^ cells was not due to a greater capacity of these cells to support infection by this virus because the same virus pseudotyped with the vesicular stomatitis virus glycoprotein (VSV-G) demonstrated no such preference (Fig 6B). Further, CCR5-tropic envelopes had a significantly greater propensity to target CD4^high^ progenitors compared to CXCR4 and dual-tropic envelopes (Fig 6B and 6C). Thus, relative CD4 expression levels correlated with susceptibility of HSPCs to infection by HIV-1 and HSPCs that express higher levels of CD4 are more likely to become infected.

### Characterization of CD4^+^CD133^+^ and CD34^+^ cells

**Fig 7:**
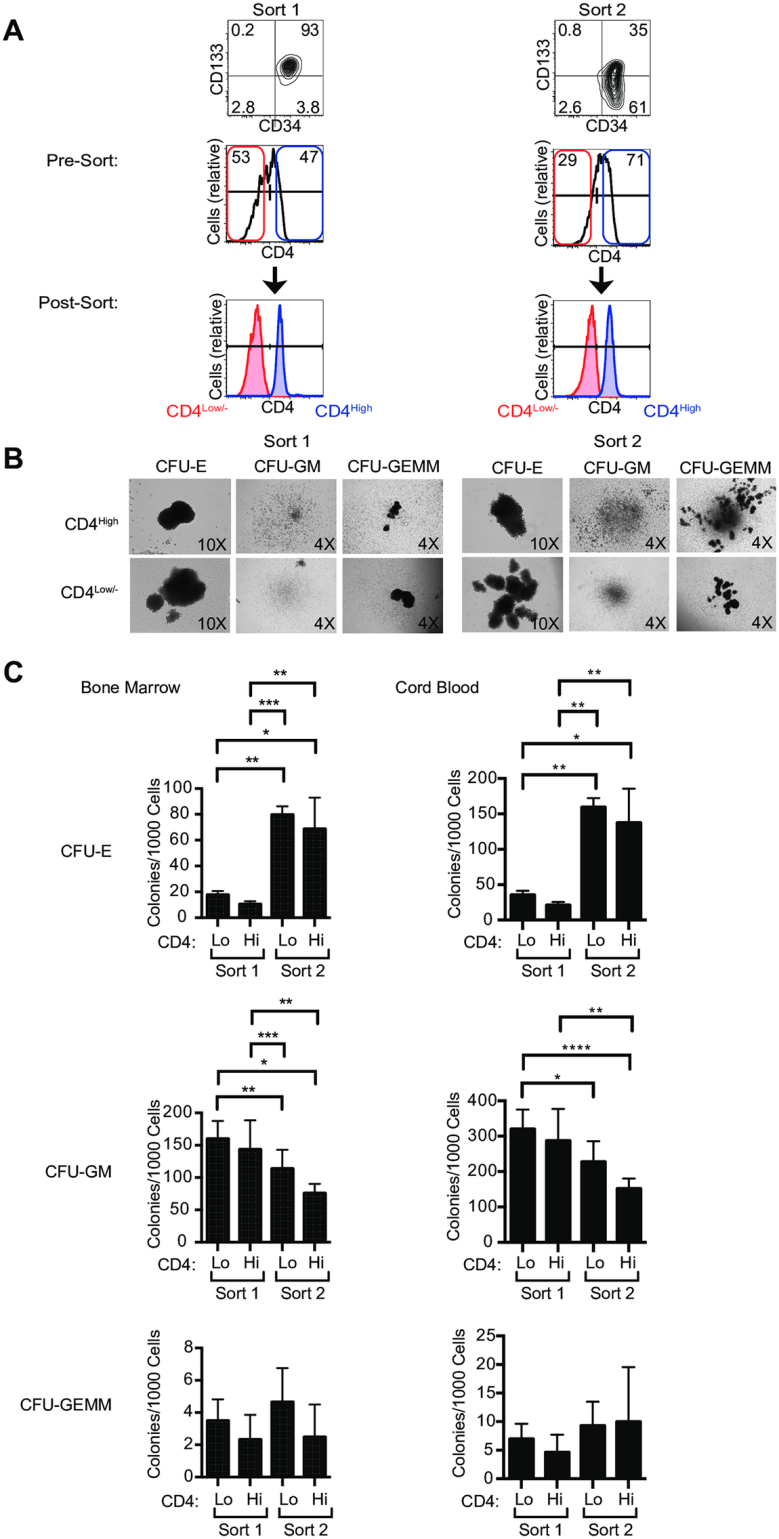
CD4^high^ HSPCs include progenitors with multi-lineage potential. A. Representative flow plots of Sort 1 and Sort 2 bone marrow-derived HSPCs sorted using fluorescence activated cell sorting into CD4^high^ and CD4^low/-^ subsets. B and C. Sorted HSPCs were analyzed by methylcellulose colony formation assays. Mean ± standard deviation is indicated for counts from three blinded investigators; 2-tailed Student’s t-test (*p<0.05, **p<0.01, ***p<0.001, ****p<0.0001). CFU-E, erythroid; CFU-GM, granulocyte/macrophage and CFU-GEMM, multilineage.

To determine whether CD4 marks a stable and separable progenitor subset with unique characteristics, we used flow cytometry to determine whether HSPCs could be separated into low and high CD4 expressing cells. Remarkably, sorting separated two distinct HSPC populations with different levels of CD4 (Fig 7A). We then used these populations to demonstrate that CD4^high^ HSPCs could form GEMM, granulocyte/macrophage (GM), and erythroid (E) colonies (Fig 7B). Thus, CD4 marks a subset of HSPCs that includes a number of different types of progenitors, including multipotent progenitors capable of generating multi-lineage GEMM colonies.

**Fig 8:**
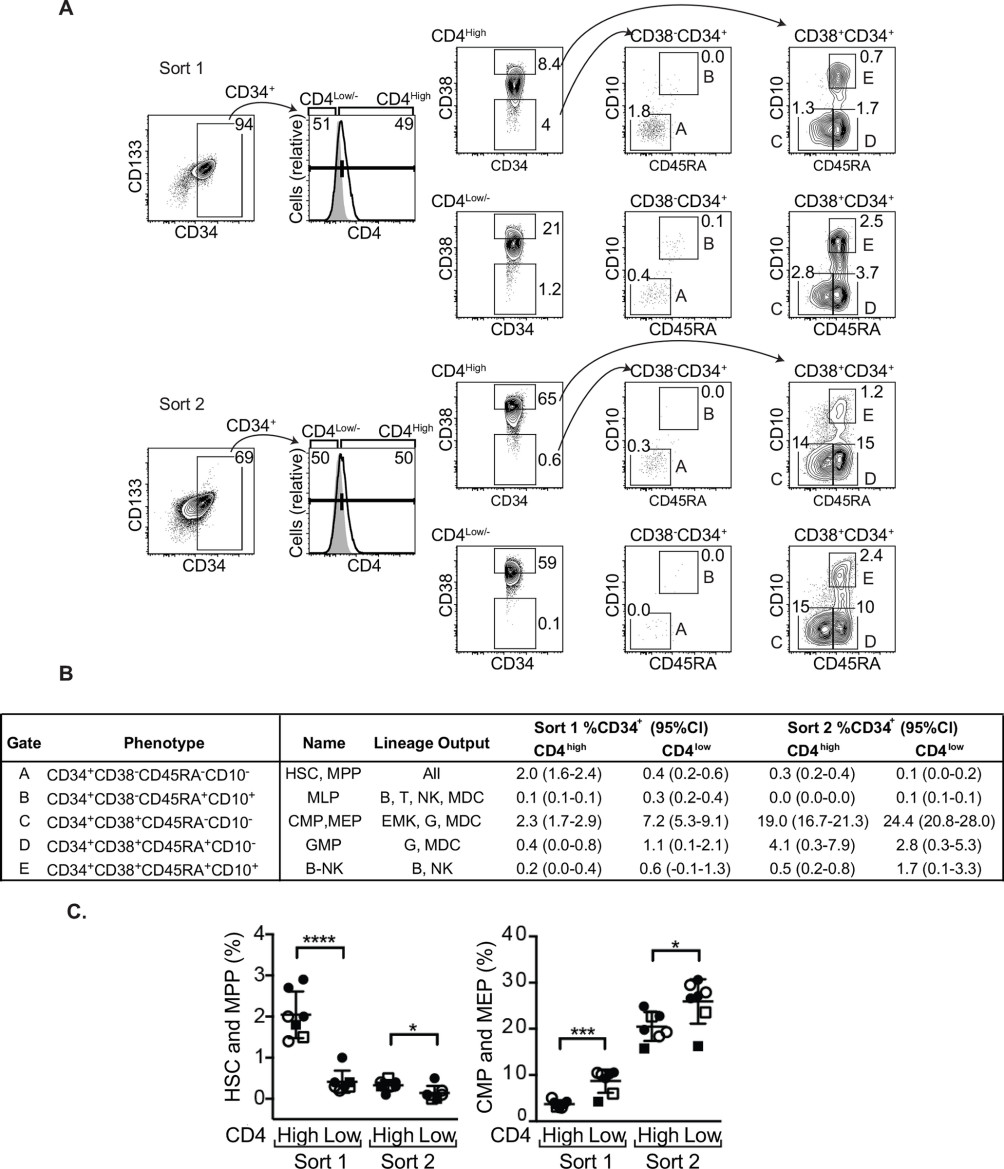
CD4^high^ HSPCs include progenitors with multi-lineage potential. A. Flow cytometric analysis of differentiation markers expressed on bone marrow HSPCs purified as described in Fig 1A. For the two right-most panels, numbers indicate percentage of total CD34^+^ events in each sort falling into that gate. B. Summary table of frequencies for each phenotypic gate as shown in A. Lineage outputs based upon Doulatov *et al* [9]. (Abbreviations: HSC, hematopoietic stem cell; MPP, multipotent progenitor; MLP, multilymphoid progenitor; CMP, common myeloid progenitor; MEP, megakaryocyte/erythrocyte progenitor; GMP, granulocyte/monocyte progenitor; B-NK, B and NK cell progenitor; MDC, macrophage and dendritic cell; EMK, erythroid and megakaryocyte) C. Summary graphs depicting the percentage of each subset of the total CD34^+^ cells in each sort. Cells were isolated from cord blood (n = 5, circles) or bone marrow (n = 2, squares). For three experiments (2 cord blood and 1 bone marrow), lineage-positive cells were physically or analytically excluded from analysis (open symbols). Mean ± standard deviation is indicated; 2-tailed Student’s t-test (*p<0.05, ***p<0.001, ****p<0.0001).

To examine the CD4^high^ sub-population in more detail, we used cell surface markers that had been validated with functional assays for HSPC subsets [9]. Remarkably, we found that CD4^high^ HSPCs in Sort 1 contained a significantly greater frequency of HSCs and MPPs (CD38^-^CD10^-^CD45RA^-^) than CD4^low^ HSPCs in the same Sort (Fig 8). Because CD133 also marks populations enriched for HSCs, we confirmed this result by demonstrating that there were significantly higher levels of CD4 on CD133^high^ HSPCs than on CD133^dim^ HSPCs (ratio paired t test, *p* = 0.020). In contrast, Sort 1 CD4^low/-^ HSPCs and all Sort 2 cells that had lower levels of CD133 (including those that were relatively CD4^high^) were less frequently HSC/MPPs and more frequently restricted progenitors such as CMP/MEPs (Fig 8). Similar results were obtained whether or not lineage positive cells were depleted from the sample prior to analysis (Fig 8C, open symbols). Thus, CD4 is expressed by a heterogeneous subset of hematopoietic progenitors and is expressed at significantly higher levels on subsets that include HSCs and MPPs.

### Transmission of HIV provirus from progenitor cells to CD4-negative progeny in vivo by proliferation and differentiation

**Fig 9:**
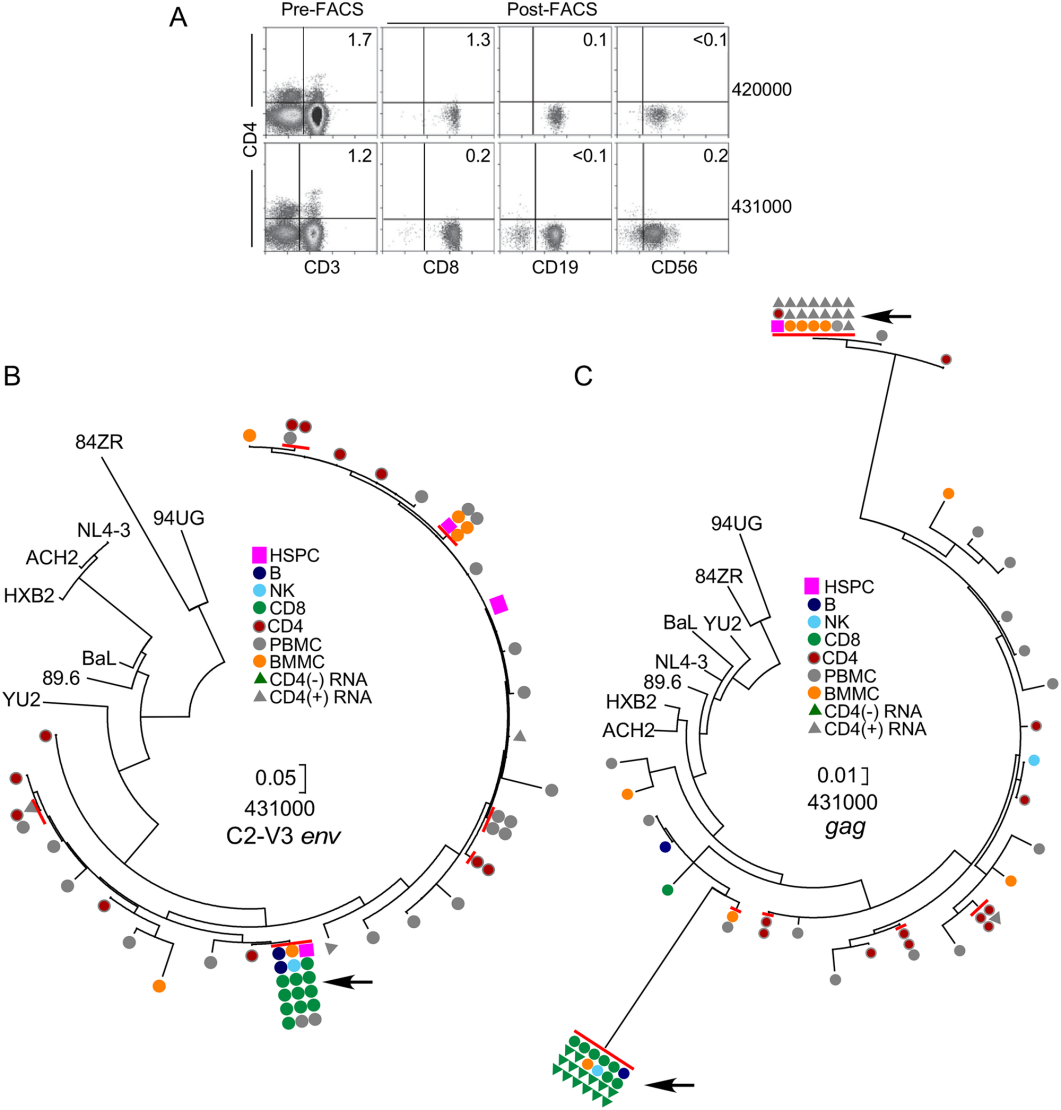
Evidence for transmission of proviral genomes from multipotent CD4^+^ HSPCs to differentiated peripheral blood cells. A. Flow cytometric plots showing purity of CD4-negative lineages containing provirus identical to HSPC-derived provirus. “Pre” indicates the cell population post CD4-bead depletion and prior to fluorescence activated cell sorting (FACS). “Post” indicates the cell populations following FACS. Numbers in the upper right corner indicate the frequency of cells in that quadrant. The frequency of CD4^+^ cells that were also CD3^+^ by gating was 0% (see also Table 5). B and C. Phylogenetic trees showing genetic relationships amongst amplicons. HIV RNA shown is cell-associated (Fig 10B). Arrows indicate location of identical amplicons shown in Fig 10. Red lines indicate identical sequences. Scale indicates nucleotide substitutions per site. ACH2, 89.6, BaL, YU-2, HXB2 and NL4-3 are subtype B HIVs. 84ZR085 (84ZR) and 94UG114 (94UG) are subtype D HIV molecular clone outgroups [32]. Phylogenetic analysis was performed by maximum likelihood method using MEGA7[33] and history was inferred based on the Hasegawa-Kishino-Yano model [34]. The tree with the highest log likelihood is shown. Abbreviations: PBMC, unfractionated peripheral blood mononuclear cells; BMMC, bone marrow mononuclear cell (column flow-through).

If HIV infects progenitor cells in vivo, HIV genomes could theoretically be passed to differentiated daughter cells as long as differentiation did not lead to reactivation of the virus from latency and cell death. To determine whether HIV can be transmitted by differentiation of infected progenitors, we assessed HIV proviral frequency in CD4-negative HSPC progeny. (CD4-negative progeny were chosen for this analysis because cells lacking this HIV receptor are unlikely to be directly infected.) To reduce the possibility of contamination by CD4-expressing cells, we depleted CD4^+^ cells using an anti-CD4 magnetic bead column prior to fluorescence activated cell sorting (FACS). Following bead depletion and FACS, CD3^+^CD4^+^ T cells were undetectable in most samples (Table 5). Moreover, lineage-positive cells (CD19^+^ B cells, CD8^+^ T cells and CD56^+^ natural killer (NK) cells) were >98% CD4 negative (indicated as “post-FACS” in Fig 9A).

**Table 5:** HIV isolation from CD4-negative lineages from a subset of ten donors.

		CXCR4					No CXCR4				
	Donor:	409	413	420	428	432	415	419	431	434	435
CD8	CD8^+^ (%)	100	100	97	99	97	97	99	96	98	98
	CD3^+^CD4^+^ (%)	0.0	0.0	0.0	0.0	0.0	0.0	0.0	0.0	0.0	0.0
	Cells Analyzed (x10^4^)	100	100	100	90	100	100	100	158	100	100
	Rxn	20	20	20	18	20	20	20	40	20	20
	LTR-gag	0	1*	3*	2****	1*	0	0	1****,7****	0	0
	C2-V3	0	0	1* (X4)	0	0	0	0	11****# (R5)	0	0
B	CD19^+^ (%)	97	97	99	98	77	96	84	87	98	94
	CD3^+^CD4^+^ (%)	0.0	0.0	0.0	0.0	0.0	0.0	0.1	0.0	0.0	0.1
	Cells Analyzed (x10^4^)	100	90	140	140	82	100	44	93	100	100
	Rxn	20	20	30	36	20	20	20	20	25	33
	LTR-gag	0	0	0	0	0	0	0	1**,1**	0	0
	C2-V3	0	0	0	0	0	0	0	2**# (R5)	0	0
NK	CD56^+^ (%)	78	96	89	93	63	84	84	80	96	95
	CD3^+^CD4^+^ (%)	0.1	0.0	0.0	0.0	0.0	0.0	0.1	0.0	0.0	0.0
	Cells Analyzed (x10^4^)	99	100	120	63	54	100	62	100	100	62
	Rxn	20	20	21	20	16	20	20	22	20	20
	LTR-gag	0	0	2*#	0	0	0	0	1**,1**	0	0
	C2-V3	0	0	0	0	2** (X4)	0	0	1*# (R5)	0	0

Asterisks indicate the likelihood the amplicon did not come from contaminating CD3^+^CD4^+^ T cell DNA. *p* values were determined as described in Fig 2.

*p< 0.05 by (1) only

**p< .05 by (1) and (2)

****p< .001 by (1) and (2) where (2) is the more conservative analysis.

^#^ indicates amplicons identical to HSPC amplicons

Grey highlights detected provirus and underlines indicate amplicons identical to other CD4-negative amplicons from the same donor.

LTR-*gag* or C2-V3 refers to amplicon type.

Rxn indicates number of reactions performed.

To determine whether HIV proviral DNA was present in these lineages, we used multiplex SGA PCR as described above. Remarkably, we generated a total of 38 LTR-*gag* or C2-V3*env* amplicons from four of five donors with CXCR4-tropic HIV but only one of five donors with only CCR5-tropic virus (Table 5). In two cases (donors 420000 and 431000), amplicons were identical to those isolated from HSPCs (indicated as # in Table 5). These cells were highly purified with undetectable CD3^+^CD4^+^ T cells (Fig 9A and Table 5). Using a quantitative statistical analysis, we found that the amplicons from CD4-negative lineages were unlikely to have come from contaminating CD3^+^CD4^+^ T cells (*p*<0.05-p<0.001, Table 5). These results provide, strong evidence that HIV provirus can be transmitted from infected progenitors to progeny cells in vivo.

**Fig 10:**
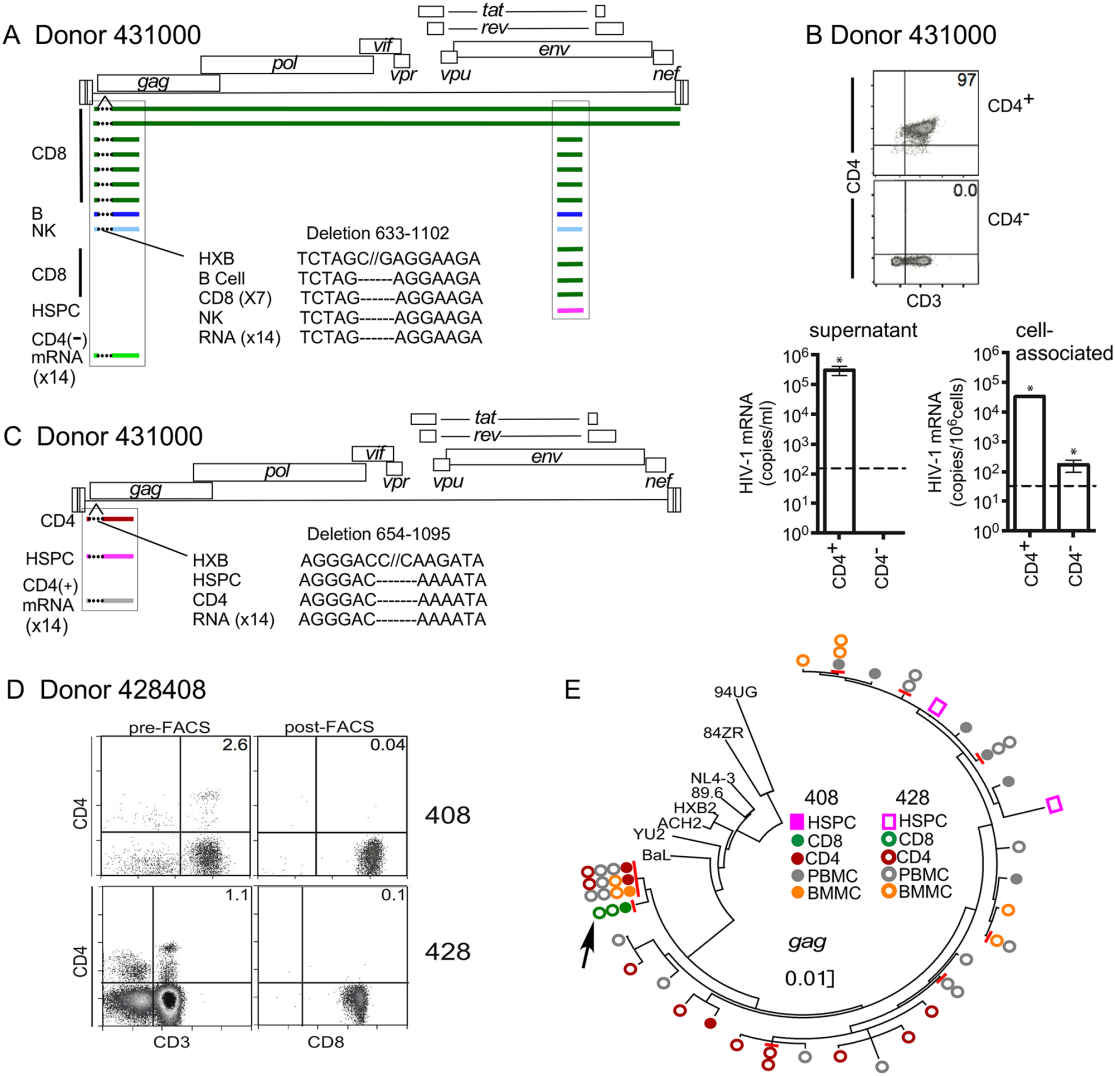
Identical HIV proviral genomes in CD4-negative progeny. A and C. Diagrammatic representation of clonal, defective proviral genomes from the indicated donor 431000 cell types. Colored bars indicate regions of identity. Dotted lines indicate location of deletion. White space indicates region where no sequence information is available. mRNA is cell associated from part B. B, (upper panel) Flow cytometric analysis of purified CD4-negative and CD4^+^ populations isolated from donor 431000 PBMCs. (Lower panels) Summary graphs of HIV-1 RNA isolated from supernatant (left) and cells (right) following treatment with PMA and ionomycin. Dashed line indicates limit of detection. *p<0.05 D and E. Provirus found in CD4-negative progeny can be recovered in two independent donations separated by months. D. Flow cytometric plots showing purity of CD4-negative lineages. “Pre” indicates the cell population post CD4-bead depletion and prior to fluorescence activated cell sorting (FACS). “Post” indicates the cell populations following FACS. Numbers in the upper right corner indicate the frequency of cells in that quadrant. The frequency of CD4^+^ cells that were also CD3^+^ by gating was 0% (see also Table 5). E. Phylogenetic tree showing genetic relationships amongst amplicons from two separate donations (408 and 428). Arrow indicates location of identical 693 bp *gag* amplicon from CD8 cells. (The region of identity extended through the non-translated LTR region of the amplicon, which was not included in the phylogenetic analysis.) Red lines indicate identical sequences. Scale indicates nucleotide substitutions per site. 89.6, BaL, YU-2, HXB2 and NL4-3 are subtype B HIVs. 84ZR085 (84ZR) and 94UG114 (94UG) are subtype D HIV molecular clone outgroups [32]. Phylogenetic analysis was performed by maximum likelihood method using MEGA7 [33] and history was inferred based on the Hasegawa-Kishino-Yano model [34]. The tree with the highest log likelihood is shown. Abbreviations: PBMC, unfractionated peripheral blood mononuclear cells; BMMC, bone marrow mononuclear cell (column flow-through).

Although we only detected provirus in CD4-negative cells from one of five donors (431000) with predominantly CCR5-tropic HIV, this donor provided the strongest evidence for HIV infection of multi-potent progenitors. Indeed, using SGA PCR, we amplified 14 identical CCR5-tropic C2-V3*env* amplicons from all three CD4-negative lineages, which were perfect matches to one another as well as to an amplicon isolated from HSPCs [Figs 9B and 10A]. In addition, seven first round SGA multiplex PCR reactions generated both C2-V3*env* amplicons as well as LTR-*gag* amplicons, all of which were identical (Table 5, Figs 9C and 10A). Remarkably, these amplicons contained a signature 469 bp deletion that removed the packaging site, the major splice site and the *gag* start codon, effectively inactivating the virus (Fig 10A). We confirmed that the deleted *gag* came from the same proviral genome as the C2-V3*env* amplicons by using SGA PCR to isolate two near-full-length genome amplicons from CD4-negative cells (Fig 10A). The presence of replication defective clonal proviral genomes in multiple differentiated hematopoietic lineages and in HSPCs provides strong evidence that infected multi-potent progenitors persist and differentiate in optimally treated people.

A phylogenetic analysis of all donor sequences ensured that all donor 431 sequences clustered together, ruling out contamination and cross contamination as confounding factors (S1 Fig). Moreover, phylogenetic analysis revealed that amplicons isolated from CD4-negative cells (B, NK and CD8) were not common in CD4^+^ cells or unfractionated PBMCs, making cross-contamination an unlikely explanation for their relatively high frequency in CD4-negative lineages (Fig 9B and 9C). Further, we used previously described statistical analysis [10] to demonstrate that the LTR-*gag* amplicons from B and NK cells were unlikely to have come from contaminating CD8^+^ T cells [p<0.05 (1)].

#### CD4-negative lineages containing a deleted provirus transcribe RNA containing the deletion but cannot produce infectious virus

To confirm that the provirus described in Fig 9 was inactive, and could not have spread to other cells through viral infection, we separated CD4-negative and positive cells (Fig 10B, upper panels), activated them with PMA and ionomycin and measured virus released into the supernatant. As shown in Fig 10B, lower left panel, CD4-negative peripheral blood cells harboring the defective provirus did not generate detectable virus in the supernatant following stimulation ex vivo, whereas CD4^+^ cells did. However, HIV RNA containing the identical deleted sequence shown in 10A was isolated from the CD4-negative cells (Fig 10B, lower right panel), confirming they contained the defective provirus (Figs 9C and 10A). Demonstration that the provirus in these cells was defective rules out the possibility that they were coincidentally infected with similar viruses and indicates that they most likely received the provirus via differentiation from a common progenitor cell.

#### CD4-positive cells also harbored a unique deleted provirus derived from a distinct HSPC

CD4^+^ cells tested in parallel also generated HIV mRNA but not the same sequences found in the CD4-negative cells. In addition to intact *gag* amplicons, one of the most prominent mRNA species amplified from CD4^+^ cells contained a unique deletion that was not found in CD4-negative cells and exactly matched provirus amplified from an HSPC (Figs 9C and 10C). In sum, these results provide evidence that HSPCs can transmit proviral genomes to both CD4^+^ and CD4-negative cells through differentiation in vivo.

#### Proviral genomes isolated from CD4-negative hematopoietic lineages persist over time

Finally, we isolated identical 831 bp 5’LTR-*gag* amplicons from CD4-negative CD8 cells from two donations separated in time by about four months (Fig 10D and 10E). While these amplicons clustered with other sequences from this donor (S1 Fig), they were not identical to any other sequences (Fig 10E, arrow). Compared to the nearest group of similar sequences, there were 11 additional differences or a genetic distance of 0.12. Thus, these data demonstrate that clonal sequences from CD4-negative lineages can be repeatedly isolated from the same cell type and persist over time in vivo, providing confirmatory evidence that pathways for infection of CD4-negative cells via infection of CD4^+^ progenitors exist in vivo.

## Discussion

The identification and characterization of cell types harboring HIV genomes is crucial for the development of strategies to promote clearance. HSPCs support both active and latent infection by HIV in vitro and in vivo [13, 18]. However, prior studies suggested a model in which only CXCR4-tropic viruses, which infect long-lived HSCs would be capable of persisting in vivo [3]. Here, we provide evidence that non-stem cell CD34^+^ progenitors infected by CCR5-tropic viruses are also long-lived. Indeed, HIV provirus isolated from HIV-infected people treated with cART for years was often CCR5-tropic and recoverable from HSPC populations that were depleted for stem cells. These unexpected results support recent studies showing that non-stem cell progenitors can persist in vivo for years and provide evidence that they may form a significant reservoir in HIV infected people.

We also provide strong evidence that progenitor cells, including multipotent progenitors, harbor HIV receptors. These results are consistent with other studies investigating the lineage potential of CD4 subsets using functional assays [16, 17, 19]. Two studies showed that CD34^+^ CD4^high^ and CD4^low/-^ populations include clonogenic progenitors and Louache *et al* furthermore demonstrated that CD34^+^ CD4^+^ HSPCs are enriched for long-term culture-initiating cells [16, 17]. Another study extended these results using human fetal liver to show that CD34^+^CD4^+^ cells are able to engraft in an immunodeficient mouse, unlike CD34^+^CD4^-^ cells [19]. In addition, HIVs that require CD4 for entry are able to infect and express marker genes in HSCs based on a gold standard functional assay (stable engraftment and generation of all hematopoietic lineages) [3]. Thus, CD4 and other HIV receptors are expressed on hematopoietic progenitors.

Preferential infection of the CD4^high^ subset partially explains another study that was unable to detect provirus in HSPCs from infected people [20]. In this study, flow cytometry was used to isolate Lin^−^CD34^+^ CD4^-^ cells, obtaining a mean purity of 76.7% that was substantially lower than the samples described here (mean purity 94.1% for Sort 1 and 90.3% for Sort 2). Based on the data presented here, removal of the CD4^+^ population would have removed the HSPC population most likely to be infected. In addition, this small study of 8 donors (3 initiating therapy during chronic infection and 5 initiating therapy during acute infection) was underpowered to detect provirus in HSPCs. The authors estimate that in these 8 patients, if proviral genomes were present, their frequency would be 0.0003%–0.003% (upper 95% confidence bounds). Given that 59% of our donors were positive and that the mean frequency of provirus in our cohort was 2.4 copies per million cells (0.0002%; range 18 to < 0.8 copies per million cells), the small study size and the small number of cells screened provide additional explanations for why this and another similarly powered study [21] were negative.

Importantly, we isolated four near full-length genomes from HSPCs and a detailed analysis of open reading frames and cis-acting elements revealed they are likely to be functional. However, demonstration of functional virus using viral outgrowth assays will require additional studies using larger cell numbers. Studies in T cells have shown that only about a tenth of functional virus can be detected in outgrowth assays [22]. A Poisson analysis using a mean frequency of 3 copies of provirus per million HSPCs with 30% functional based on sequencing suggests 60 million HSPCs will be needed for 95% certainty of detecting one infectious unit. Given that we obtain about 2.5 million HSPCs for each donor from 20 cc of marrow, we would need to dramatically increase our aspiration size to acquire sufficient cells, which would not be easy to accomplish because of patient discomfort. The low rate of infection in HSPCs likely explains why an earlier study utilizing low numbers of HSPCs (approximately one million) yielded negative results in outgrowth assays [21]. In addition, while we have shown that transcriptionally latent viral genomes in HSPCs can be reactivated by TNFα and histone deacetylase inhibitors in vitro after cell culture [3, 13], studies using large cell numbers are needed to determine the optimal strategies to effectively reactivate proviral genomes to promote viral release from fully quiescent HSPCs tested ex vivo.

Nevertheless, the conclusion that HIV indeed infects HSPCs was confirmed by the detection of clonal HIV proviral genomes in differentiated lineages that matched provirus from HSPCs. Because the differentiated cells were CD4-negative lineages and because the provirus contained signature inactivating deletions, these results can’t be explained by coincident infection. Moreover, we confirmed the presence of these genomes by isolating cell-associated mRNA containing the same deletion from activated CD4-negative cells. Further, we showed by phylogenetic analysis that the genomes frequently isolated from CD4-negative lineages formed a unique clonal population within the donor, indicating that contamination from other cell types was an unlikely explanation of our findings. In sum, the most likely explanation is that these genomes were transmitted to CD4-negative progeny through differentiation of a CD4-positive progenitor. In addition, we also detected a proviral genome with a unique signature deletion in both HSPC and CD4^+^ cells indicating that infected HSPCs can also differentiate into CD4^+^ cells.

In most cases, detection of proviral genomes in CD4-negative lineages was rare with only a small number of proviral genomes detected per million cells screened. The exception was donor 431000 in which we detected a defective provirus at a higher frequency (approximately one per 100,000 cells screened). Because replication competent virus could disrupt differentiation due to cytopathic effects, it is not surprising that viral spread from differentiating HSPCs would be uncommon with functional virus, occurring at a higher frequency in cells harboring a defective viral genome that might allow normal differentiation to occur.

In addition, we detected proviral genomes more often in CD4-negative lineages from donors with CXCR4-tropic virus, consistent with its ability to target a wider range of HSPC subtypes, including MPPs and HSCs. With the exception of one donor (431000), we did not find CCR5-tropic provirus in differentiated CD8, B and NK lineages found in the peripheral blood, which is consistent with observations that CCR5-tropic HIV more commonly infects restricted myeloid progenitor cells [4].

Although HSCs are the main drivers for reconstitution of all hematopoietic lineages in xenograft models, new insights in animal and human disease models have shown contributions of non-stem cell progenitors to steady state hematopoiesis over long periods of time [6–8]. Non-stem cell progenitors appear to survive longer than previously thought in the bone marrow without contribution from HSCs, with non-stem cell clones sequentially recruited over time to produce mature blood cells [6–8, 23, 24]. Our data that CCR5-tropic provirus persists for years in non-stem cell progenitors is to our knowledge the first evidence that non-stem cell progenitors persist for years in humans without evidence of bone marrow disease.

Given that non-stem cell progenitors persist, the prevalence of CCR5-tropic HIV in this compartment is not surprising. During acute infection when circulating virus peaks, the majority of virus is CCR5-tropic [25]. However, we also detected persistent provirus that encodes Env proteins capable of utilizing CXCR4 to enter cells. Assuming transmitting virus is nearly uniformly CCR5-tropic, as some studies have indicated, the presence of persistent reservoirs of CXCR4-tropic provirus may indicate that reservoirs continue to form during evolution to CXCR4 tropism in some donors.

Overall, these results support a new model in which non-stem cell progenitors are important long term contributors to normal hematopoiesis and moreover that these cells can serve as a persistent reservoir for HIV provirus.

## Materials and methods

### Ethics statement

HIV-infected individuals were recruited through the University of Michigan HIV-AIDS Treatment Program and the Henry Ford Health System. Written informed consent was obtained according to a protocol approved by the University of Michigan Institutional Review Board and Henry Ford Institutional Review Board (U-M IRB number HUM00004959 and HFH IRB number 7403). Donors were >18 years old, with normal white blood cell counts and plasma viral loads were <48 copies/ml for at least 6 months on antiretroviral therapy. 100 ml of peripheral blood and 20 ml of bone marrow were obtained from each donor. All collected samples were coded.

Whole umbilical cord blood (CB) from uninfected donors was obtained from the New York Blood Center and whole bone marrow was obtained commercially (AllCells Ltd.). All collected samples were anonymized.

### Cell isolation and fractionation

For isolation of HSPCs, mononuclear cells were purified by Ficoll-Hypaque centrifugation and adherence depleted in serum-free StemSpan medium (StemCell Technologies) for 1–2 hours at 37°C. Sort 1 cells were isolated with a CD133 MicroBead Kit (Miltenyi Biotec) according to the manufacturer’s protocol, using two sequential sorts for increased purity. (For donations 453000, and 454304, we used 1.5 times the recommended MicroBeads to increase yield.) Sort 2 cells were isolated from the Sort 1 flow-through using EasySep Human CD34 Positive Selection Kit (StemCell Technologies) according to the manufacturer’s protocol, using two sequential sorts. Where indicated, lineage-positive cells were depleted using the EasySep Lineage Depletion Kit (StemCell Technologies) before proceeding to the CD133 magnetic sort.

CD4 negative PBMCs from the human donors described in the ethics statement were purified by depletion of CD4^+^ cells with MicroBeads (Miltenyi Biotec) according to the manufacturer’s protocol modified for a bead:cell ratio of 1.5:1 and passage over two sequential LS magnetic columns. Depleted cells were stained and sorted as indicated in the text to remove residual CD4^+^ cells on a MoFlo Astrios flow cytometer.

### RNA isolation

Supernatant and cell associated RNA was extracted using TRIzol LS and TRizol reagents, respectively according to the manufacturer’s protocols (Invitrogen) and converted to cDNA using qScript cDNA Supermix or qScript Flex cDNA Kit according to manufacturer’s instructions (Quanta Biosciences). RNA from viral supernatants was quantified by real time PCR using TaqMan Fast Mastermix (Applied Biosystems) on an Applied Biosystems 7300 thermocycler using primers and probes as previously described [26] and used in SGA PCR described below.

For gene expression analysis, bone marrow cells were isolated and harvested as described [27]. RNA was extracted from 3x10^4^ double-sorted cells from each cell population. RNAseq was performed on the total RNA extracted from each cell population, adding equal amounts of 92 spiked-in RNA standards to each cell population. Since the amount of spiked-in RNA standards added to each sample was known, the relationship between RPKM (reads per kilobase per million) values and the number of transcripts for each spiked-in RNA could be determined by regression analysis [28]. RNAseq reads were aligned using Bowtie software [29] to NCBI build 37 (mm9) of the mouse genome with the settings: -e 70 -k 1 -m 2—n 2. The RPKM for each RefSeq gene and synthetic spike-in RNA was calculated using RPKM_count.py (v2.3.5) counting only exonic reads (-e option). Loess regression from R affy package was used to renormalize the RPKM values by using only the spike-in RNA to fit the loess with default parameters. Only the spike-in RNAs whose abundance could be robustly quantified (RPKM values ≥ 1) were used in the loess normalization.

### SGA PCR of patient samples

DNA was prepared using the MagNA Pure Compact System (Roche) and used at limiting dilution for a 2-step SGA PCR validated for single copy sensitivity on ACH-2 cell DNA using primer sets shown in S5 Table. PBMC DNA from each donor was used to select the optimal primer sets for each donor. PCR assays were performed using a BioRad C1000 thermocycler as described in S6 Table. Amplicons were sequenced directly from the purified gel band.

### Virus infection

Virus was prepared by transfection of HIV or lentiviral genome containing plasmids into 293T (ATCC) cells as described [13]. Where indicated, the helper plasmid pCMV-HIV-1[30] and a plasmid encoding either VSV-G protein or an HIV envelope protein were used as described previously [3, 31]. Intracellular Gag staining was performed as previously described [13].

### Determination of Env phenotype

*Env* expression vectors were generated using gel purified DNA cloned into pcDNA3.1/V5-His-TOPO TA. For phenotypic analysis of donor Env matching the HSPC V3 region, we used 3T3-CD4-CCR5 and CXCR4 cells (NIH AIDS Reagent Repository) [11] transduced with HIV-7/SF-GFP pseudotyped with the each Env and harvested for flow cytometry 3 days post-transduction.

### Antibodies used for flow cytometry

Antibodies to the following human proteins were used for flow cytometry: CD133 (phycoerythrin [PE] conjugated; Miltenyi Biotec), CD34 (conjugated with fluorescein isothiocyanate [FITC], allophycocyanin [APC], PE-Cy7; Miltenyi Biotec and eBioscience), CD3 (APC; eBioscience or APC-H7-conjugated; BD Bioscience), CD4 (clone OKT4, unconjugated, Brilliant Violet 605 conjugated; BD Biosciences or AlexaFluor488 conjugated; eBioscience), CD45RA (APC conjugated; eBioscience), CD38 (PE-Cy7 conjugated; eBioscience), CD10 (Biotin conjugated; eBioscience), HIV-1 Gag (clone KC57, FITC conjugated; Beckman Coulter), CD8 (PE conjugated; BioLegend), CD19 (APC conjugated; BD Bioscience), CD56 (PE-Cy7 conjugated; eBioscience).and Human Hematopoietic Lineage Cocktail (FITC conjugated; eBioscience). The secondary reagents used were streptavidin (Brilliant Violet 421 conjugated; BD Biosciences) and anti-mouse IgG2b (Alexa Fluor 647 conjugated; Invitrogen). Non-viable cells were identified and excluded from sorts and analyses by staining with 7-aminoactinomycin D (7-AAD) or DAPI. Samples were analyzed using a BD FacsCanto cytometer. Cell sorting was performed using a MoFlo XDP (Beckman Coulter), MoFlo Astrios (Beckman Coulter), FACSJazz (BD Biosciences) or FACSAria (BD Biosciences) flow cytometer.

For flow cytometric analysis and isolation of specific murine hematopoietic progenitors for RNA isolation, cells were incubated with combinations of antibodies to the following cell-surface markers conjugated to FITC, PE, PerCP-Cy5.5, APC, PE-Cy7, or biotin: CD3ε (17A2), CD4 (GK1.5), CD5 (53–7.3), CD8α (53–6.7), CD11b (M1/70), CD16/32 (FcΥRII/III; 93), CD34 (RAM34), CD43 (1B11), CD45R (B220; RA3-6B2), CD48 (HM48-1), CD117 (c-kit; 2B8), CD127 (IL7Rα; A7R34), CD150 (TC15-12F12.2), Ter119 (TER-119), Sca1 (D7, E13-161.7), Gr-1 (RB6-8C5), and IgM (II/41). For isolation of CD150^+^CD48^-^Lineage^-^Sca-1^+^c-kit^+^ (CD150^+^CD48^-^LSK) HSCs and CD150^-^CD48^-^LSK MPPs, lineage markers included CD3, CD5, CD8, B220, Gr-1, and Ter119. For isolation of CD34^+^CD16/32^low^CD127^-^Sca-1^-^LK CMPs and CD34^+^CD16/32^high^CD127^-^Sca-1^-^LK GMPs, these lineage markers were supplemented with antibodies against CD4 and CD11b. Other sorted populations included Gr-1^+^ cells, IgM^-^CD43^+^B220^+^ pro-B cells, IgM^-^CD43^-^B220^+^ pre-B cells, and unfractionated bone marrow cells.

## Supporting information

S1 Fig Phylogenetic trees showing genetic relationships amongst donor *gag* and *env* (C2-V3) amplicons. 89.6, BaL, YU-2, HXB2, ACH2 and NL4-3 are subtype B HIVs. 84ZR085 (84ZR) and 94UG114 (94UG) are subtype D HIV molecular clone outgroups [32].
(TIF)

S2 Fig Frequency of HIV proviral genomes in purified HSPCs does not correlate with CD3^+^ T cell frequency. Linear regression analysis showing the lack of a correlation between CD3^+^ T cell contamination and proviral frequency (R square values were 0.05 and 0.0008 for Sort 1 and Sort 2 respectively). The number of cells analyzed for purity assessment for each donor is summarized in S4 Table. (To ensure rigor, samples containing >1% CD3^+^ T cells were excluded.)
(TIF)

S3 Fig CD3^+^ T cell contamination can’t explain HIV amplicons isolated from purified HSPCs from donor 413402. Agarose gel analysis of rearranged TCRβ amplicons generated from 5000 cell equivalents of DNA from first round PCR reactions [35]. The expected size of the amplicons is 250–300 bp and the faint band in Sort 1 lane 7 could not be verified as TCRβ by sequencing analysis. NT, No template; 293, control human embryonic kidney cell line (1000 cells); M, DNA ladder. Numbers indicate number of CD4^+^ T cells added to positive control reactions. * indicates samples containing HIV DNA based on PCR analysis. The overall frequency of HIV DNA detected in the flow-through sample from this donor was approximately 1 per 10,000 cells.
(TIF)

S1 Table HSPC purity and assessment of T cell contamination for multiplex *gag/C2env* SGA PCR. **P values shown indicate the likelihood that amplicons did not originate from contaminating T cell DNA. *p* values were determined either using a mean cell estimate or a conservative estimate as in McNamara et al (ref. 10). The conservative estimate compared the top of the 95% confidence interval for the calculated infection rate in CD3^+^ T cells in the HSPC-depleted sample with the bottom of the 95% confidence interval for the calculated infection rate in CD3^+^ T cells in the HSPC-sorted sample to minimize the difference between these calculated infection rates. *First 3 digits is donation number; subsequent groups of 3 digits are ID of previous donation(s) from the same individual, if any. Bold borders indicate multiple donations from the same individual. Gray boxes indicate samples that did not meet criteria for purity based on CD3%>1.0 or <80% HSPC (CD34 or CD133). Abbreviations: NA, not analyzed.
(PDF)

S2 Table C2-V3 amplicon screen of first round reactions using whole genome primers and analysis of T cell contamination. **P values shown indicate the likelihood that amplicons did not originate from contaminating T cell DNA. *p* values were determined either using a mean cell estimate or a conservative estimate as in McNamara et al (ref. 10). The conservative estimate compared the top of the 95% confidence interval for the calculated infection rate in CD3^+^ T cells in the HSPC-depleted sample with the bottom of the 95% confidence interval for the calculated infection rate in CD3^+^ T cells in the HSPC-sorted sample to minimize the difference between these calculated infection rates. *First 3 digits is donation number; subsequent groups of 3 digits are ID of previous donation(s) from the same individual, if any. Bold borders indicate multiple donations from the same individual. Gray boxes indicate samples that did not meet criteria for purity based on CD3%>1.0 or <80% HSPC (CD34 or CD133). Abbreviations: NA, not analyzed.
(PDF)

S3 Table Cis elements in donor near-full-length genomes. Amplicons correspond to HXB2 positions 604–9599 and “-”indicates region not amplified. “Y” indicates identity to HXB2, red indicates differences and lower case denotes insertions. pbs, tRNA primer binding site; SL, packaging stem loop; DIS, dimerization initiation site; MSD, major splice donor. (Sequences and locations from HIV Sequence Compendium 2015, Los Alamos National Laboratory.).
(PDF)

S4 Table Number of cells analyzed by flow for purity. (PDF)

S5 Table PCR primer sequences. (PDF)

S6 Table PCR cycling conditions. *Conditions optimized for 1^st^ round of multiplex PCR with *gag* primers (U5-577.9662-f plus tagD4.6b-p24R1d plus or minus long1316-D4.6b).
(PDF)
