# Correction: CD4 is expressed on a heterogeneous subset of hematopoietic progenitors, which persistently harbor CXCR4 and CCR5-tropic HIV proviral genomes in vivo

**DOI:** 10.1371/journal.ppat.1006617

**Published:** 2017-09-13

**Authors:** The PLOS Pathogens Staff

There are two errors in Table 3. The purity values (98, 98) should be listed below donor ID 409000. In addition, in the first column of the 409000 donor ID row, the third line should read 1*. The publisher apologizes for the errors.

**Table 3:** Analysis of *env* amplicons isolated from HIV^+^ donors.

Donor ID (% purity) (S1, S2)	HSPC Sort 1 (S1) *env* amplicons				HSPC Sort 2 (S2) *env* amplicons				PBMC *env* amplicons		
	#	FPR	Geno^b^	Pheno^c^	#	FPR	Geno	Pheno	#	FPR	Geno
409000 (98, 98)	1****w 1****w 1*	61 0.7 0.7	R5 X4/Dual X4/Dual		ND				17 11	38–61 0.7	R5 X4/Dual
413402 (94, 91)	1*	1.7	X4/Dual	Dual	ND				6	1.7	X4/Dual
414000 (95, 85)	1*	57	R5	R5	ND				5	57	R5
415000 (95, NA)	1**	71	R5	R5	NA				20	43–83	R5
419000 (95, NA)	1***	89	R5		NA				21	22–99	R5
420000 (99, 92)	1**** 1****w 1**** 1****w	19 73 0.7 8.1	R5 R5 X4/Dual X4/Dual	R5 Dual	ND				11 7	14–73 0.5–1.7	R5 X4/Dual
421000 (99, 96)	1***w 2***	7.8 7.4,7.8	X4/Dual X4/Dual	X4	1**w	7.8	X4/Dual		4 5	24–94 4.7–9.6	R5 X4/Dual
426000 (97, 92)	ND				4**** 2****w 1****w	29–60 20–31 4.8	R5 R5 X4/Dual		10 1	20–38 3.2	R5 X4/Dual
428408 (95, 86) (91, 91)	2**** 1****	83,84 1.3	R5 X4/Dual		1**w	1.3	X4/Dual		22 16	30–100 0.7–6.8	R5 X4/Dual
431000 (93, 89)	2**** 1****	75 79	R5 R5	R5 R5	ND				8	38–90	R5
432000 (98, 99)	1**** 1****	17 2.8	R5 X4/Dual	X4	1**** 1****	2.8 3.4	X4/Dual X4/Dual	NF	1 8 1 11 4	49 3.4–8.5 74 2.8–4.7 3.4–8.5	R5 X4/Dual R5 X4/Dual X4/Dual
434423 (92, 89) (94, 84)	1**	38	R5		1**	46	R5		18	38–82	R5
435412406 (99, 99) (98, 83) (95, 94)	1** 2**** 1** 1**w	83 87 89 45	R5 R5 R5 R5	R5 R5 R5	1**	83	R5	R5	103	42–99	R5
436000 (93, 85)	ND				2**	31	R5	R5	8	31–85	R5
437000 (94, 92)	1**	55	R5				ND		8	12–55	R5
449000 (90, 95)	1*	100	R5		1**	81	R5		5	59–99	R5
453000 (96, 83)	ND				1***	74	R5	R5	6	41–86	R5
454304 (92,93)	ND				1* 1*w	31 9.6	R5 X4/Dual		24 1	11–97 6.8	R5 X4/Dual
456000 (93,90)	ND				3***	13–39	R5		10 2	10.5–39 7.4, 9.2	R5 X4/Dual

^#^ indicates the number of amplicons of that type isolated from each donor.

^b^genotypic prediction of co-receptor usage by Geno2pheno using a false positive rate (FPR) cutoff of _10% [36, 37].

^c^phenotypic analysis of co-receptor usage (see Table 4).

“w” indicates amplicons from whole genome PCR and black text indicates amplicons from multiplex PCR reaction.

Asterisks reflect the likelihood that CD3+ T cells contamination was responsible for the amplicon

*p < 0.05 by (1) only

**p < .05 by (1) and (2)

***p < .01 by (1) and (2)

****p < .001 by (1) and (2) where (2) is using the more conservative assessment (Fig 2) [10].

Abbreviations: HSPC, hematopoietic stem and progenitor cells; PBMC, peripheral blood mononuclear cells; NA, not analyzed due to purity concerns; R5, CCR5; X4, CXCR4; ND, not detectable; NF, non-functional.

Each set of three non-zero numbers in the donor name represents an independent donation.
