# Supplementary figures and images for: CD4 is expressed on a heterogeneous subset of hematopoietic progenitors, which persistently harbor CXCR4 and CCR5-tropic HIV proviral genomes in vivo
Source: PLoS Pathog. 2017 Jul 21;13(7):e1006509. doi: 10.1371/journal.ppat.1006509 (PMC5540617; doi:10.1371/journal.ppat.1006509)

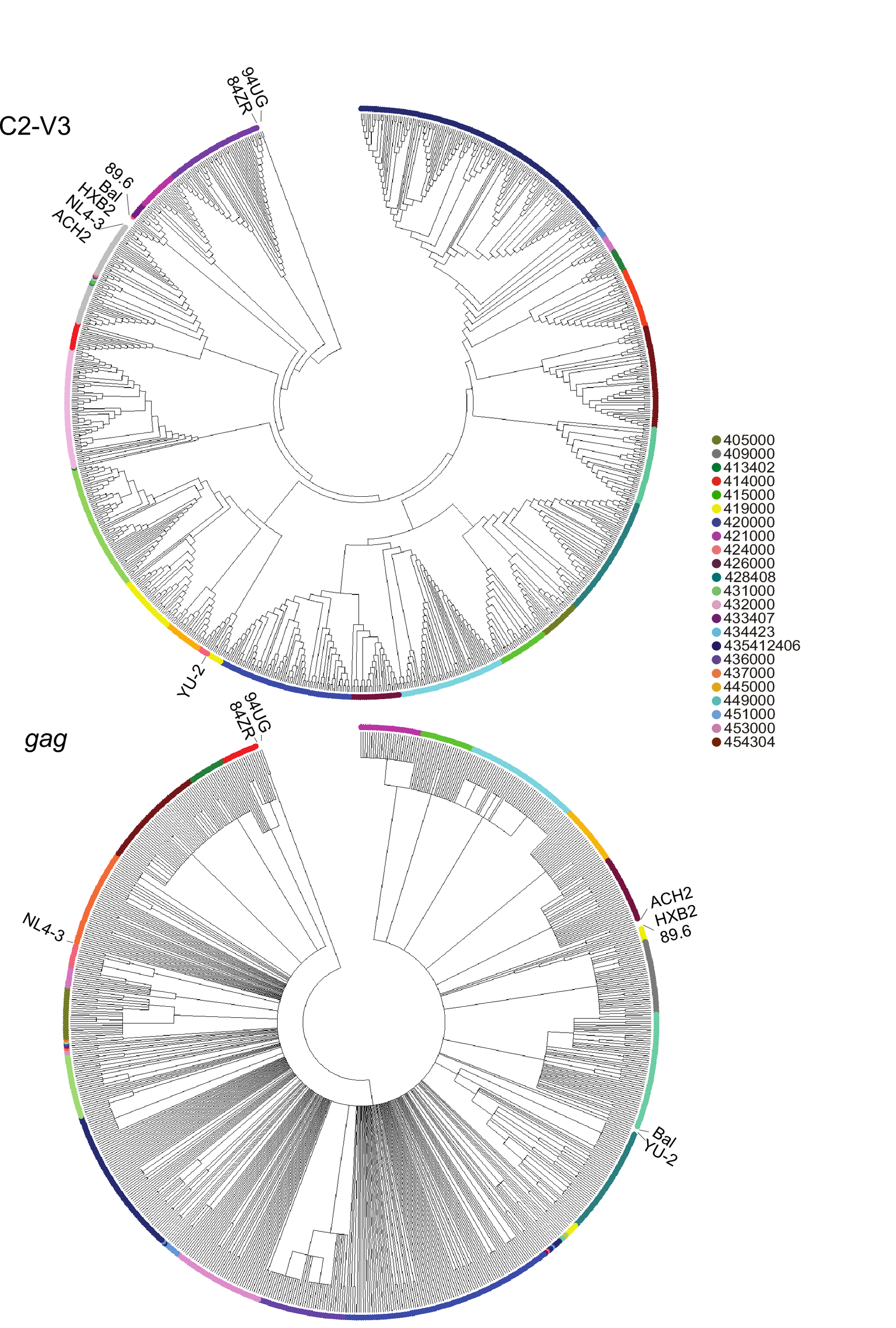

Supplement: S1 Fig — 89.6, BaL, YU-2, HXB2, ACH2 and NL4-3 are subtype B HIVs. 84ZR085 (84ZR) and 94UG114 (94UG) are subtype D HIV molecular clone outgroups [32]. (TIF) [file ppat.1006509.s001.tif]

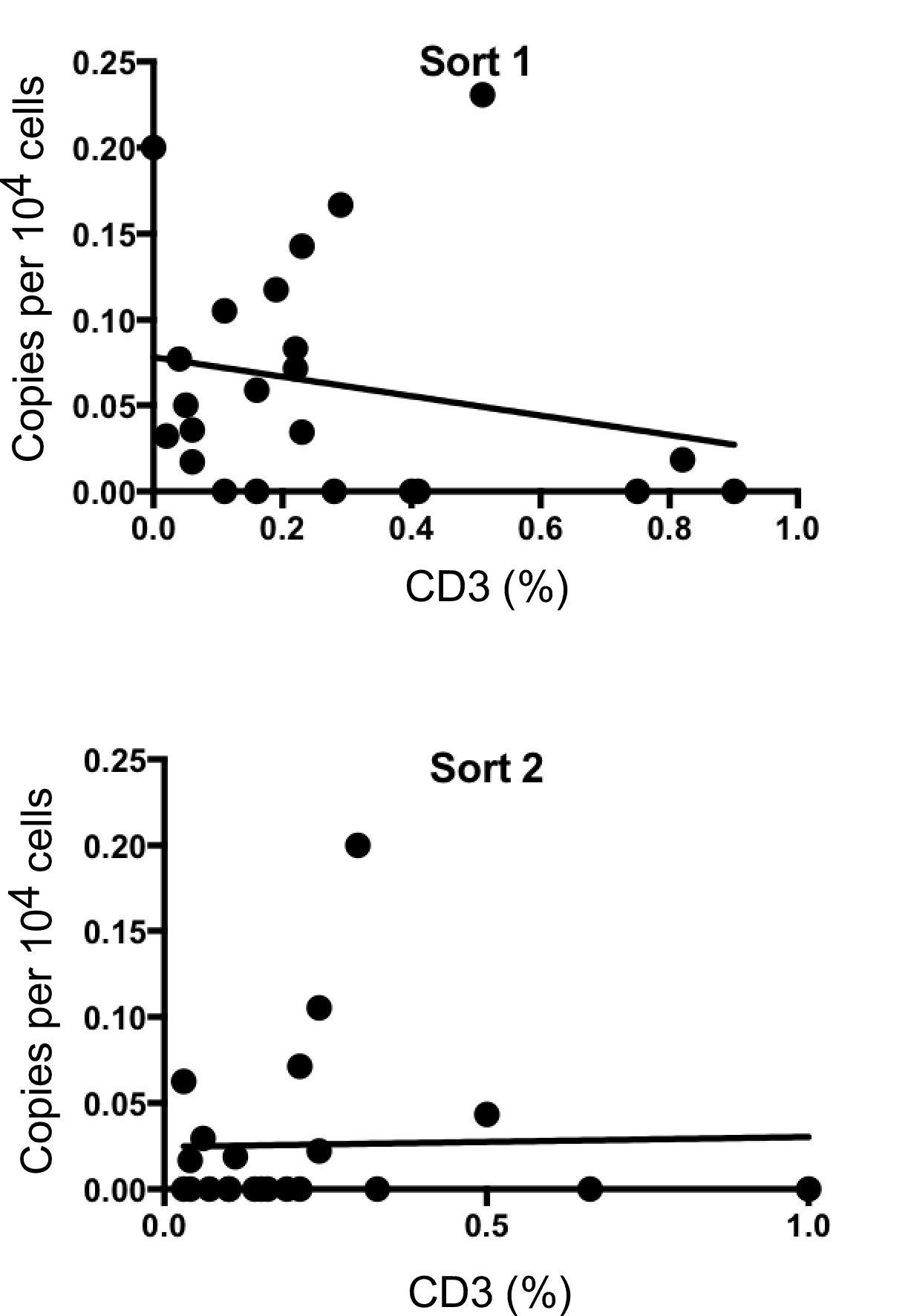

Supplement: S2 Fig — Linear regression analysis showing the lack of a correlation between CD3+ T cell contamination and proviral frequency (R square values were 0.05 and 0.0008 for Sort 1 and Sort 2 respectively). The number of cells analyzed for purity assessment for each donor is summarized in S4 Table. (To ensure rigor, samples containing >1% CD3+ T cells were excluded.) (TIF) [file ppat.1006509.s002.tif]

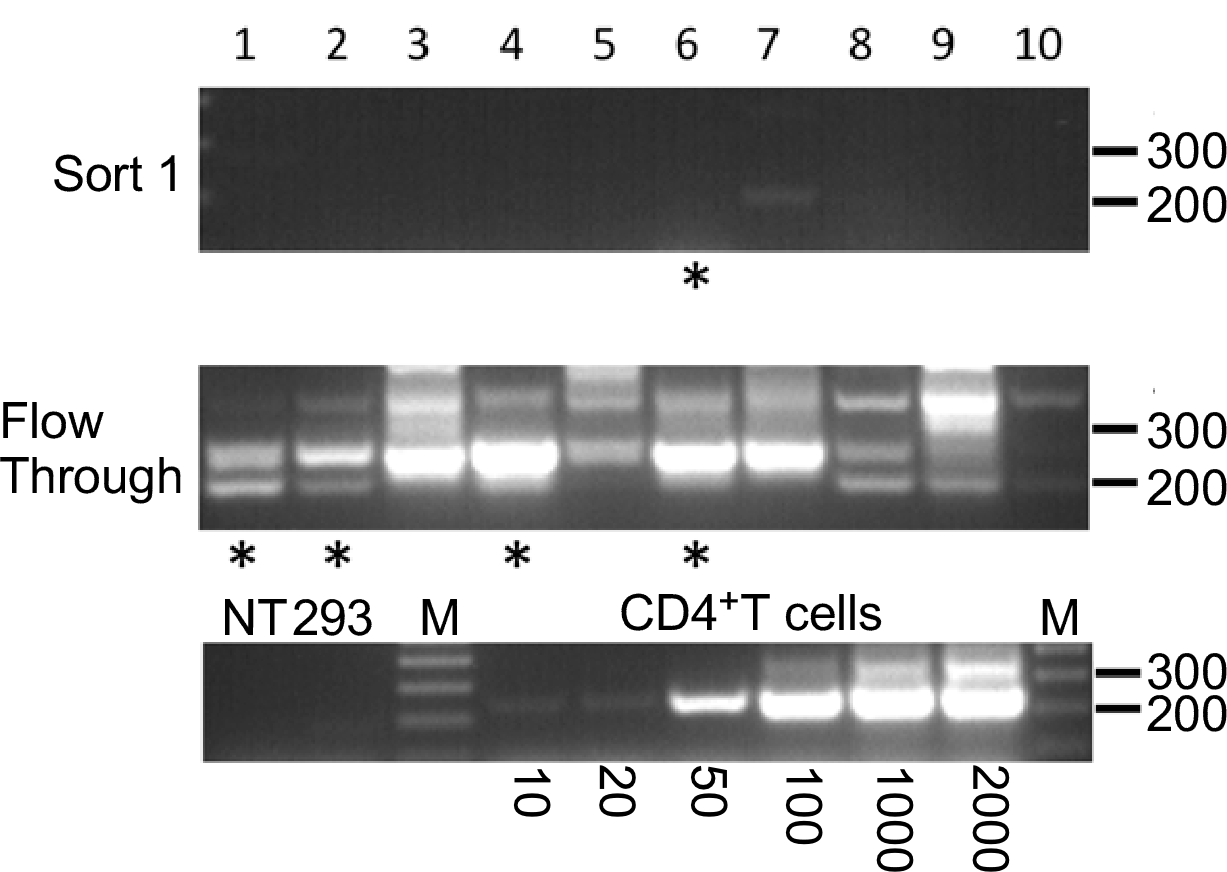

Supplement: S3 Fig — Agarose gel analysis of rearranged TCRβ amplicons generated from 5000 cell equivalents of DNA from first round PCR reactions [35]. The expected size of the amplicons is 250–300 bp and the faint band in Sort 1 lane 7 could not be verified as TCRβ by sequencing analysis. NT, No template; 293, control human embryonic kidney cell line (1000 cells); M, DNA ladder. Numbers indicate number of CD4+ T cells added to positive control reactions. * indicates samples containing HIV DNA based on PCR analysis. The overall frequency of HIV DNA detected in the flow-through sample from this donor was approximately 1 per 10,000 cells. (TIF) [file ppat.1006509.s003.tif]
