# Supplementary material for: CD4 is expressed on a heterogeneous subset of hematopoietic progenitors, which persistently harbor CXCR4 and CCR5-tropic HIV proviral genomes in vivo
Source: PLoS Pathog. 2017 Jul 21;13(7):e1006509. doi: 10.1371/journal.ppat.1006509 (PMC5540617; doi:10.1371/journal.ppat.1006509)
Supplement: S1 Table — **P values shown indicate the likelihood that amplicons did not originate from contaminating T cell DNA. p values were determined either using a mean cell estimate or a conservative estimate as in McNamara et al (ref. 10). The conservative estimate compared the top of the 95% confidence interval for the calculated infection rate in CD3+ T cells in the HSPC-depleted sample with the bottom of the 95% confidence interval for the calculated infection rate in CD3+ T cells in the HSPC-sorted sample to minimize the difference between these calculated infection rates. *First 3 digits is donation number; subsequent groups of 3 digits are ID of previous donation(s) from the same individual, if any. Bold borders indicate multiple donations from the same individual. Gray boxes indicate samples that did not meet criteria for purity based on CD3%>1.0 or <80% HSPC (CD34 or CD133). Abbreviations: NA, not analyzed. (PDF) [file ppat.1006509.s004.pdf]

Donation ID*	Sort 1				Flowthrough 1			Due to CD3+ T Cell Contamination?**		Sort 2				Flowthrough 2			Due to CD3+ T Cell Contamination?	
	CD133$^+$ (%)	CD3$^+$ (%)	V3 Amplicons	Cells Analyzed (x10^4)	CD3$^+$ (%)	V3 Amplicons	Cells Analyzed (x10^4)	p value (Mean Estimate)	p value (Conservative Estimate)	CD34$^+$ (%)	CD3$^+$ (%)	V3 Amplicons	Cells Analyzed (x10^4)	CD3$^+$ (%)	V3 Amplicons	Cells Analyzed (x10^4)	p value (Mean Estimate)	p value (Conservative Estimate)
409000	98	0.11	1	9.5	55	3	2.8	2.6E-02	6.3E-02	98	0.07	0	11	61	5	4.7	NA	NA
413402	94	0.0	1	5.0	49	1	1.7	5.0E-04	1.1E-01	91	0.19	0	19	53	4	3.1	NA	NA
414000	95	0.22	1	14	57	1	3.0	3.7E-02	6.6E-02	85	0.33	0	27	53	1	9.2	NA	NA
415000	96	0.06	1	28	40	1	4.6	1.7E-02	4.0E-02	72	0.48	NA	NA	33	3	23	NA	NA
419000	96	0.02	1	31	56	3	31	1.3E-03	4.5E-03	78	0.06	NA	NA	52	3	35	NA	NA
420000	99	0.04	2	26	37	4	19	3.2E-05	1.8E-04	92	1.0	0	41	29	8	19	NA	NA
421000	99	0.19	2	17	53	4	9.4	6.0E-04	1.6E-03	96	0.10	0	37	50	3	33	NA	NA
426000	97	0.28	0	20	44	2	17	NA	NA	92	0.24	4	38	50	1	38	2.5E-09	1.4E-08
408000	95	NA	0	2.0	NA	2	0.60	NA	NA	86	NA	0	3.0	NA	1	1.0	NA	NA
428408	91	0.51	3	13	47	6	13	1.0E-04	3.9E-04	91	0.16	0	5.5	47	12	7.8	NA	NA
431000	93	0.23	3	21	28	5	21	2.7E-05	2.1E-04	89	0.15	0	26	27	2	16	NA	NA
432000	98	0.05	2	40	18	7	30	4.0E-04	1.6E-03	99	0.03	2	32	20	5	32	3.5E-05	2.3E-04
423000	92	0.06	1	58	54	4	23	1.5E-02	3.8E-02	89	0.03	0	70	54	10	34	NA	NA
434423	94	0.16	0	67	30	0	16	NA	NA	84	0.11	1	53	30	2	53	1.1E-02	1.8E-02
406000	99	0.29	2	12	62	1	1.6	3.3E-03	1.6E-02	99	0.14	0	20	57	1	3.2	NA	NA
412406	96	0.40	0	20	51	1	1.8	NA	NA	83	0.06	1	34	61	1	3.8	1.7E-02	4.5E-02
435412406	95	0.22	2	24	40	3	24	3.1E-04	6.5E-04	94	0.66	0	56	40	7	31	NA	NA
436000	93	0.90	0	6.1	52	2	3.6	NA	NA	85	0.30	2	10	53	6	11	7.2E-04	2.4E-03
437000	94	0.16	1	17	16	0	17	9.9E-03	1.6E-02	92	0.04	0	43	18	1	43	NA	NA
449000	90	0.23	1	29	32	6	29	5.0E-02	7.0E-02	95	0.04	1	60	33	14	60	1.9E-02	4.5E-02
453000	96	0.75	0	41	61	0	16	NA	NA	83	0.24	1	45	62	0	45	3.9E-03	6.5E-03
454304	92	0.41	0	12	47	2	12	NA	NA	93	0.21	1	14	46	7	14	3.6E-02	9.1E-02
456000	93	0.76	0	68	72	2	14	NA	NA	90	0.31	3	67	70	10	67	2.5E-05	6.8E-05

**P values shown indicate the likelihood that amplicons did not originate from contaminating T cell DNA. p values were determined either using a mean cell estimate or a conservative estimate as in McNamara et al (ref. 10). The conservative estimate compared the top of the 95% confidence interval for the calculated infection rate in CD3+ T cells in the HSPC-depleted sample with the bottom of the 95% confidence interval for the calculated infection rate in CD3+ T cells in the HSPC-sorted sample to minimize the difference between these calculated infection rates. *First 3 digits is donation number; subsequent groups of 3 digits are ID of previous donation(s) from the same individual, if any. Bold borders indicate multiple donations from the same individual. Gray boxes indicate samples that did not meet criteria for purity based on CD3%>1.0 or <80% HSPC (CD34 or CD133). Abbreviations: NA, not analyzed
