# Supplementary material for: CD4 is expressed on a heterogeneous subset of hematopoietic progenitors, which persistently harbor CXCR4 and CCR5-tropic HIV proviral genomes in vivo
Source: PLoS Pathog. 2017 Jul 21;13(7):e1006509. doi: 10.1371/journal.ppat.1006509 (PMC5540617; doi:10.1371/journal.ppat.1006509)
Supplement: S2 Table — **P values shown indicate the likelihood that amplicons did not originate from contaminating T cell DNA. p values were determined either using a mean cell estimate or a conservative estimate as in McNamara et al (ref. 10). The conservative estimate compared the top of the 95% confidence interval for the calculated infection rate in CD3+ T cells in the HSPC-depleted sample with the bottom of the 95% confidence interval for the calculated infection rate in CD3+ T cells in the HSPC-sorted sample to minimize the difference between these calculated infection rates. *First 3 digits is donation number; subsequent groups of 3 digits are ID of previous donation(s) from the same individual, if any. Bold borders indicate multiple donations from the same individual. Gray boxes indicate samples that did not meet criteria for purity based on CD3%>1.0 or <80% HSPC (CD34 or CD133). Abbreviations: NA, not analyzed. (PDF) [file ppat.1006509.s005.pdf]

| Donation ID* | Sort 1 | | | | Flowthrough 1 | | | Due to CD3+ T Cell Contamination?** | | Sort 2 | | | | Flowthrough 2 | | | Due to CD3+ T Cell Contamination?** | |
|---|---|---|---|---|---|---|---|---|---|---|---|---|---|---|---|---|---|---|
| | $CD133^+$ (%) | $CD3^+$ (%) | V3 Amplicons | Cells Analyzed ($x10^4$) | $CD3^+$ (%) | V3 Amplicons | Cells Analyzed ($x10^4$) | p value (Mean Estimate) | p value (Conservative Estimate) | $CD34^+$ (%) | $CD3^+$ (%) | V3 Amplicons | Cells Analyzed ($x10^4$) | $CD3^+$ (%) | V3 Amplicons | Cells Analyzed ($x10^4$) | p value (Mean Estimate) | p value (Conservative Estimate) |
| 409000 | 98 | 0.11 | 2 | 9.5 | 55 | 1 | 2.8 | 1.3E-04 | 7.8E-04 | 98 | 0.07 | ND | 43 | 61 | NA | NA | | |
| 419000 | 96 | 0.03 | ND | 31 | 56 | NA | NA | | | 78 | 0.06 | ND | 35 | 52 | NA | NA | | |
| 420000 | 99 | 0.04 | 2 | 26 | 37 | 5 | 26 | 2.3E-05 | 1.3E-04 | 92 | 1 | 1 | 41 | 29 | 3 | 12 | 3.6E-01 | 4.4E-01 |
| 421000 | 99 | 0.19 | 1 | 16 | 53 | ND | 7.7 | 7.1E-03 | 1.2E-03 | 96 | 0.1 | 1 | 37 | 50 | 2 | 32 | 6.8E-03 | 1.6E-02 |
| 426000 | 97 | 0.28 | ND | 20 | 44 | NA | NA | | | 92 | 0.24 | 3 | 38 | 50 | 1 | 37 | 4.9E-07 | 1.8E-06 |
| 428408 | 91 | 0.51 | ND | 1.7 | 47 | NA | NA | | | 91 | 0.16 | 1 | 10 | 47 | ND | 5.0 | 6.8E-03 | 1.1E-02 |
| 431000 | 93 | 0.23 | ND | 1.3 | 28 | NA | NA | | | 89 | 0.15 | ND | 0.8 | NA | NA | NA | | |
| 423000 | 92 | 0.06 | NA | NA | 54 | NA | NA | | | 89 | 0.03 | ND | 20 | 54 | NA | NA | | |
| 406000 | 99 | 0.29 | ND | 12 | 62 | NA | NA | | | 99 | 0.14 | ND | 20 | 57 | NA | NA | | |
| 412406 | 96 | 0.4 | 1 | 13 | 51 | ND | 1.8 | 5.3E-02 | 8.0E-02 | 83 | 0.06 | ND | 34 | 61 | NA | NA | | |
| 435412406 | 95 | 0.22 | 1 | 19 | 40 | 1 | 19 | 1.1E-02 | 1.6E-02 | 94 | 0.66 | ND | 45 | 40 | NA | NA | | |
| 436000 | 93 | 0.9 | NA | NA | 52 | NA | NA | | | 85 | 0.3 | ND | 0.2 | 53 | NA | NA | | |
| 437000 | 94 | 0.16 | ND | 17 | 16 | NA | NA | | | 92 | 0.04 | ND | 43 | 18 | NA | NA | | |
| 449000 | 90 | 0.23 | ND | 29 | 32 | NA | NA | | | 95 | 0.04 | ND | 60 | 33 | NA | NA | | |
| 453000 | 96 | 0.75 | ND | 41 | 61 | NA | NA | | | 83 | 0.24 | ND | 45 | 62 | NA | NA | | |
| 454304 | 92 | 0.41 | ND | 9.6 | 47 | NA | NA | | | 93 | 0.21 | 1 | 14 | 46 | 4 | 14 | 2.3E-02 | 5.8E-02 |

**P values shown indicate the likelihood that amplicons did not originate from contaminating T cell DNA. p values were determined either using a mean cell estimate or a conservative estimate as in McNamara et al (ref. 10). The conservative estimate compared the top of the 95% confidence interval for the calculated infection rate in CD3+ T cells in the HSPC-depleted sample with the bottom of the 95% confidence interval for the calculated infection rate in CD3+ T cells in the HSPC-sorted sample to minimize the difference between these calculated infection rates. *First 3 digits is donation number; subsequent groups of 3 digits are ID of previous donation(s) from the same individual, if any. Bold borders indicate multiple donations from the same individual. Gray boxes indicate samples that did not meet criteria for purity based on CD3%>1.0 or <80% HSPC (CD34 or CD133). Abbreviations: NA, not analyzed
