# Supplementary material for: CD4 is expressed on a heterogeneous subset of hematopoietic progenitors, which persistently harbor CXCR4 and CCR5-tropic HIV proviral genomes in vivo
Source: PLoS Pathog. 2017 Jul 21;13(7):e1006509. doi: 10.1371/journal.ppat.1006509 (PMC5540617; doi:10.1371/journal.ppat.1006509)
Supplement: S3 Table — Amplicons correspond to HXB2 positions 604–9599 and “-”indicates region not amplified. “Y” indicates identity to HXB2, red indicates differences and lower case denotes insertions. pbs, tRNA primer binding site; SL, packaging stem loop; DIS, dimerization initiation site; MSD, major splice donor. (Sequences and locations from HIV Sequence Compendium 2015, Los Alamos National Laboratory.). (PDF) [file ppat.1006509.s006.pdf]

Feature	HXB2#		409 Sort 1-1 Position		409 Sort 1-2 Position		421 Sort 2 Position		454 Sort 2 Position	
TCF-1 a	315-329, 9400-9414	TACTTCAAGAACTGC	- 8810-88w24	TTTTACAAGGACTGC	- 8829-8843	TTTTACAAGGACTGC	- 8817-8831	TACTACAAGGACTGC	- 8785-8799	TACTACAAAGACTGC
NFkB-II	350-359, 9435-9444	GGGACTTTCC	- 8845-8854	Y	- 8864-8873	Y	- 8874-8883	Y	- 8820-8829	Y
NFkB-I	364-373, 9449-9458	GGGACTTTCC	- 8859-8868	Y	- 8864-8873	Y	- 8888-8897	Y	- 8834-8843	Y
Sp1-III	375-386, 9460-9471	GGGAGGCGTGGC	- 8870-8880	GGGAGGCGTGAC	- 8889-8900	GGGAGGCGTGAC	- 8899-8910	GGGAGGCGTGAC	- 8846-8857	GGgAGGTGTGGC
Sp1-II	388-397, 9473-9482	TGGGCGGGAC	- 8883-8892	Y	- 8902-8911	Y	- 8912-8921	Y	- 8859-8868	Y
Sp1-I	398-408, 9483-9493	TGGGGAGTGGC	- 8893-8903	AGGGGAGTGGC	- 8912-8922	AGGGGAGTGGC	- 8922-8932	Y	- 8869-8879	Y
TATAA	427-431, 9512-9516	TATAA	- 8922-8926	Y	- 8941-8945	Y	- 8962-8966	Y	- 8898-8902	Y
TAR	453-513, 9538-9598	TGGGTCTCTCTGGTTAGACCAGATCTGAGCCTGGGAGCTCTCTGGCTAACTAGGGAACCCA	- 8948-9008	TGGGTCTCTCTAGTTAGACCAGATCTGAGCCTGGGAGCTCTCTGGCTGACTAGGGAACCCA	- 8967-9027	TGGGTCTCTCTAGTTAGACCAGATCTGAGCCTGGGAGCTCTCTGGCTGACTAGGGAACCCA	- 8987-9047	TGGGTCTCTCTGGTTAGACCAGATTTGAGCCTGGGAGCTCTCTGGCTAGCTAGGGAACCCA	- 8924-8984	TGGGTCTCTCTGGTTAGACCAGATCAGAGCCTGGGAGCTCTCTGGCTAGCTAGGGAACCCA
polyA	527-532, 9612-9617	AATAAA	- -		- -		- -		- -	
pbs stem	671-680	ACCAGAGGAG	40-49	Y	68-77	Y	68-77	Y	69-79	Y
SL1	696-730	ACTCGGCTTGCTGAAGCGCGCACGGCAAGAGGCGA	65-99	Y	93-127	Y	93-127	Y	94-128	Y
DIS	711-716	GCGCGC	80-85	Y	108-113	Y	108-113	Y	109-114	Y
SL2	735-753	CGGCGACTGGTGAGTACGC	104-122	Y	132-150	Y	132-150	Y	133-151	Y
MSD	742-745	TG|GT	111-114	Y	139-142	Y	139-142	Y	140-143	Y
SL3	764-778	GACTAGCGGAGGCTA	133-147	Y	161-175	Y	161-175	Y	162-176	Y
SL4	793-806	GGTGCGAGAGCGTC	162-175	Y	190-203	Y	190-203	Y	191-204	Y
