# Supplementary material for: CD4 is expressed on a heterogeneous subset of hematopoietic progenitors, which persistently harbor CXCR4 and CCR5-tropic HIV proviral genomes in vivo
Source: PLoS Pathog. 2017 Jul 21;13(7):e1006509. doi: 10.1371/journal.ppat.1006509 (PMC5540617; doi:10.1371/journal.ppat.1006509)
Supplement: S4 Table — (PDF) [file ppat.1006509.s007.pdf]

Donation ID*	Sort 1	Flowthrough 1	Sort 2	Flowthrough 2
409000	4,604	755	8,510	2,578
413402	456	133	1,589	475
414000	4,893	11,791	5,501	8,450
415000	8,873	7,725	3,066	11,141
419000	11,441	9,087	10,873	8,253
420000	12,684	7,928	6,937	8,543
421000	7,473	9,265	5,117	8,826
426000	7,902	21,462	8,691	18,093
408000	445	9,128	168	6,246
428408	3,715	8,769	9,498	8,352
431000	3,531	15,120	4,068	15,303
432000	17,309	18,854	16,188	18,989
423000	6,292	6,679	6,484	6,807
434423	17,759	18,520	15,681	18,906
406000	2,071	651	4,355	1,059
412406	10,228	243	6,732	1,164
435412406	12,924	16,028	11,853	16,881
436000	2,339	22,671	3,372	21,769
437000	13,247	17,052	20,665	17,116
449000	14,788	10,588	11,860	12,049
453000	5,869	2,862	6,612	4,080
454304	2,430	8,139	1,915	6,179
