# Supplementary material for: CD4 is expressed on a heterogeneous subset of hematopoietic progenitors, which persistently harbor CXCR4 and CCR5-tropic HIV proviral genomes in vivo
Source: PLoS Pathog. 2017 Jul 21;13(7):e1006509. doi: 10.1371/journal.ppat.1006509 (PMC5540617; doi:10.1371/journal.ppat.1006509)
Supplement: S5 Table — (PDF) [file ppat.1006509.s008.pdf]

Amplicon	PCR Round	Primer name	Orientation	HXB2 Location	Sequence
env	1st	5036d	Forward	5036-5059	5′-GGAYTATGGAAAACAGATGGCAGG-3’
env	1st or 2nd	LTR-pA-R	Reverse	9625-9600	5′-AGGCAAGCTTTATTGAGGCTTAAGCA-3’
env	1st or 2nd	5956d-f	Forward	5956-5983	5′-CTTAGGCATYTCCTATGGCAGGAAGAAG-3’
env	1st or 2nd	envC2F2	Forward	6950–6976	5′-CAGCACAGTACAATGTACACATGGAAT-3’
env	1st or 2nd	envC4R1	Reverse	7540-7520	5′-ATGGGAGGGGCATACATTGCT-3′
env	2nd	env1in5	Forward	7060-7081	5′-ACAATGCTAAAACCATAATAGT-3′
env	2nd	env1in3	Reverse	7530-7511	5′-CATACATTGCTTTTCCTACT-3′
gag	1st or 2nd	U5-577.9662-f	Forward	577-603	5’-GACTCTGGTAACTAGAGATCCCTCAGA-3’
gag	1st	long1316-D4.6b	Reverse	1322-1316	5’-atcttgcggcgctctgtgTGGGGTGGCTCCTTCTG-3’
gag	1st	tagD4.6b-p24R1d	Reverse	1500-1480	5’-atcttgcggcgctctgtgTGCTATGTCACTTCCCCTTGG-3’
gag	2nd	626s	Forward	626-651	5’-TCTCTAGCAGTGGCGCCCGAACAGGG-3’
gag	2nd	1294r	Reverse	1318-1294	5’-CTGATAATGCTGAAAACATGGGTAT-3’
gag	2nd	D4.6b	Reverse	N/A	5’-atcttgcggcgctctgtg-3’
gagenv	2nd	1204s	Forward	1204-1226	5’-CAGGGGCAAATGGTACATCAGGC-3’
gagenv	2nd	E30HXrc	Reverse	6471-6445	5’-CTTGTGGGTTGGGGTCTGTGGGTACAC-3’
