# Supplementary material for: CD4 is expressed on a heterogeneous subset of hematopoietic progenitors, which persistently harbor CXCR4 and CCR5-tropic HIV proviral genomes in vivo
Source: PLoS Pathog. 2017 Jul 21;13(7):e1006509. doi: 10.1371/journal.ppat.1006509 (PMC5540617; doi:10.1371/journal.ppat.1006509)
Supplement: S6 Table — *Conditions optimized for 1st round of multiplex PCR with gag primers (U5-577.9662-f plus tagD4.6b-p24R1d plus or minus long1316-D4.6b). (PDF) [file ppat.1006509.s009.pdf]

Primer 1	Primer 2	Cycling Conditions
*5036d	LTR-pA-R	98°C for 30 seconds; 35 cycles of 98°C for 10 seconds, 68° C for 10 seconds, and 72°C for 2 minutes; 72°C for 10 minutes
*5956d-f	LTR-pA-R	98°C for 30 seconds; 35 cycles of 98°C for 10 seconds, 71° C for 10 seconds, and 72°C for 2 minutes; 72°C for 10 minutes
*envC2F2	envC4R1	98°C for 30 seconds; 35 cycles of 98°C for 10 seconds, 68° C for 10 seconds, and 72°C for 25 seconds; 72°C for 5 minutes
env1in5	env1in3	98°C for 30 seconds; 40 cycles of 98°C for 10 seconds, 56° C for 10 seconds, and 72°C for 15 seconds; 72°C for 5 minutes
626s	D4.6b	98°C for 30 seconds; 35 cycles of 98°C for 10 seconds, 70° C for 10 seconds, and 72°C for 40 seconds; 72°C for 10 minutes
U5-577.9662-f	LTR-pA-R	98°C for 30 seconds; 35 cycles of 98°C for 10 seconds, 67° C for 10 seconds, and 72°C for 4 minutes; 72°C for 10 minutes
U5-577.9662-f	1294r	98°C for 30 seconds; 35 cycles of 98°C for 10 seconds, 64° C for 10 seconds, and 72°C for 40 seconds; 72°C for 5 minutes
1204s	E30HXrc	98°C for 30 seconds; 35 cycles of 98°C for 10 seconds, 66° C for 10 seconds, and 72°C for 2 minutes 40 seconds; 72°C for 10 minutes

*Conditions optimized for 1st round of multiplex PCR with *gag* primers (U5-577.9662-f plus tagD4.6b-p24R1d plus or minus long1316-D4.6b)
